# The growth response to androgen receptor signaling in ER**α**-negative human breast cells is dependent on p21 and mediated by MAPK activation

**DOI:** 10.1186/bcr3112

**Published:** 2012-02-09

**Authors:** Joseph P Garay, Bedri Karakas, Abde M Abukhdeir, David P Cosgrove, John P Gustin, Michaela J Higgins, Hiroyuki Konishi, Yuko Konishi, Josh Lauring, Morassa Mohseni, Grace M Wang, Danijela Jelovac, Ashani Weeraratna, Cheryl A Sherman Baust, Patrice J Morin, Antoun Toubaji, Alan Meeker, Angelo M De Marzo, Gloria Lewis, Andrea Subhawong, Pedram Argani, Ben H Park

**Affiliations:** 1 The Sidney Kimmel Comprehensive Cancer Center, The Johns Hopkins University School of Medicine, Baltimore, MD, USA; 2 The Whiting School of Engineering, Department of Chemical and Biomolecular Engineering, The Johns Hopkins University, Baltimore, MD, USA; 3 Laboratory of Molecular Biology and Immunology, National Institute on Aging, Baltimore MD, USA

## Abstract

**Introduction:**

Although a high frequency of androgen receptor (AR) expression in human breast cancers has been described, exploiting this knowledge for therapy has been challenging. This is in part because androgens can either inhibit or stimulate cell proliferation in pre-clinical models of breast cancer. In addition, many breast cancers co-express other steroid hormone receptors that can affect AR signaling, further obfuscating the effects of androgens on breast cancer cells.

**Methods:**

To create better-defined models of AR signaling in human breast epithelial cells, we took estrogen receptor (ER)-α-negative and progesterone receptor (PR)-negative human breast epithelial cell lines, both cancerous and non-cancerous, and engineered them to express AR, thus allowing the unambiguous study of AR signaling. We cloned a full-length cDNA of human AR, and expressed this transgene in MCF-10A non-tumorigenic human breast epithelial cells and MDA-MB-231 human breast-cancer cells. We characterized the responses to AR ligand binding using various assays, and used isogenic MCF-10A p21 knock-out cell lines expressing AR to demonstrate the requirement for p21 in mediating the proliferative responses to AR signaling in human breast epithelial cells.

**Results:**

We found that hyperactivation of the mitogen-activated protein kinase (MAPK) pathway from both AR and epidermal growth factor receptor (EGFR) signaling resulted in a growth-inhibitory response, whereas MAPK signaling from either AR or EGFR activation resulted in cellular proliferation. Additionally, p21 gene knock-out studies confirmed that AR signaling/activation of the MAPK pathway is dependent on p21.

**Conclusions:**

These studies present a new model for the analysis of AR signaling in human breast epithelial cells lacking ERα/PR expression, providing an experimental system without the potential confounding effects of ERα/PR crosstalk. Using this system, we provide a mechanistic explanation for previous observations ascribing a dual role for AR signaling in human breast cancer cells. As previous reports have shown that approximately 40% of breast cancers can lack p21 expression, our data also identify potential new caveats for exploiting AR as a target for breast cancer therapy.

## Introduction

Breast cancer is a disease in which the pathogenesis can be attributed to hormone exposure, the most notable being estrogens. Successful targeted therapies against estrogen receptor (ER)α have been developed, and this remains an active area of research. Many of these therapies directly target ERα or the ERα signaling pathway, and have been shown to be highly efficacious in treating ERα-positive breast cancers [1]. However, a significant subset of breast cancers cannot be treated by these therapies because they do not express ERα or its surrogate predictive marker of response, the progesterone receptor (PR), and/or these cancers commonly show resistance to drugs that target the ERα pathway.

Androgens are another class of sex hormones, and epidemiologic studies have supported their role in breast biology and carcinogenesis [2-4]. In fact, the androgen receptor (AR) is expressed in the vast majority of breast cancers, with some studies reporting expression of AR in up to 90% of primary tumors and 75% of metastatic lesions [5,6], although more contemporary studies suggest that the frequency of AR expression varies depending on the subtype of breast cancer (for example, ERα-positive (luminal) versus triple-negative and basal breast cancers), and other clinical and pathologic parameters [7-9]. In addition, AR expression may also affect outcomes in given subsets of breast cancer. For example, in luminal breast cancers expressing AR, the AR expression is associated with better prognosis [10-12]. Of potential clinical relevance, past studies support the notion that AR agonists may have beneficial effects in treating luminal AR-positive disease [13,14]. Approximately 10% to 20% of triple-negative breast cancers are known to express AR [15], and of particular interest is the group termed 'molecular apocrine breast cancer'. This subset of tumors has been shown to be transcriptionally regulated by AR with a luminal gene-expression profile [16,17], and both *in vitro *and *in vivo *studies using anti-androgen therapies have shown promising results [16,18,19]. Additionally, approximately 20% of HER2-positive, ERα-negative breast cancers have also been shown to express AR [7,8,20]. Thus, targeting AR may offer a potent form of hormone therapy for this group of patients, yet despite this, therapies targeting AR for breast cancer are currently not in widespread use. There are numerous reasons for this, including side-effects of masculinization and organ toxicities seen with androgen use [21]. In addition, one of the most problematic issues with androgen use for breast cancer therapy is that androgens can yield either a growth-inhibitory or cell-proliferative effect in pre-clinical models, depending on the breast cancer cell lines being studied, regardless of their ERα status [22]. Moreover, separate groups have described disparate results when examining the response of the same breast cancer cell line to a given AR ligand. This is probably due to cellular changes that can occur in continuous culture, owing to the inherent genetic instability of breast cancer cell lines [23]. However, there are several reasons why AR remains a potential target for breast cancer therapy. First, as mentioned above, a significant percentage of breast cancers (10% to 20%) are AR-positive/ERα-negative, thus providing an opportunity for hormone therapies targeting AR in this group of patients. Second, the historical success of targeting AR for prostate cancer provides a proof of principle for its use as a target in cancer therapy. Third, approximately 40% to 50% of ERα-positive breast cancers treated with conventional hormone therapies such as tamoxifen or aromatase inhibitors (AIs) will recur with drug-resistant disease, and AR-directed therapies may still be efficacious in this patient population. Interestingly, a recent study suggested that AR overexpression may be a mechanism of tamoxifen resistance [24]. Thus, despite the past experience of and caveats about targeting AR for breast cancer, developing novel therapies that target AR could have a significant influence on the treatment of this disease.

As mentioned, laboratory studies assessing the role of AR in breast cancer have been limited and conflicting. In part, this is due to the fact that most AR-positive breast cancer cell lines also express ERα and PR [7,8,25-28]. This can confound analyses of AR receptor signaling for several reasons. When co-expressed, AR and ERα have been shown to physically interact and decrease transcription of response genes [29]. Further complexity occurs due to the promiscuity of a given ligand for multiple nuclear hormone receptors. For example, along with serving as a PR ligand, the synthetic progestin medroxyprogesterone acetate can also bind to AR and function as an AR agonist [30]. Likewise, the ERα antagonist fulvestrant has been shown to downregulate AR expression, and therefore attenuate response to AR ligand [31]. Understanding AR signaling in models of human breast cells that express AR exclusively would help to elucidate the role of AR in breast cancer and further the development of targeted therapies, particularly in the setting of ERα-negative disease. However, there are few breast cancer cell lines that express AR as the sole sex-hormone receptor, and those that do exist often harbor numerous genetic anomalies that could potentially alter AR signaling. For example, the cell line MDA-MD-453 is AR-positive/ERα-negative, but this cell line also has a homozygous deletion of *TP53*, a homozygous *PTEN *missense mutation, *HER2 *amplification, and an oncogenic mutation in *PIK3CA *(Sanger Catalogue of Somatic Mutations in Cancer (COSMIC) database; http://www.sanger.ac.uk/genetics/CGP/cosmic) [32,33].

To circumvent this issue, we expressed AR in a genetically well-defined, non-tumorigenic, human breast epithelial cell line, MCF-10A. This cell line is spontaneously immortalized as a result of homozygous loss of the chromosomal region 9p, but is genetically stable [23]. Further, we previously used this cell line to generate stable ERα-expressing clones with physiologic responses to estrogen including growth stimulation by estrogen, which is blocked by tamoxifen and the induction of luminal-type genes by estrogen stimulation [34].

In this paper, we report and characterize a similar model to study AR signaling in human breast epithelial cell lines. We found that in MCF-10A cells expressing AR, co-stimulation of EGFR signaling with AR ligand binding led to a growth-inhibitory effect due to hyperactivation of the mitogen-activated protein kinase (MAPK) pathway. However, MAPK activation with either AR ligand binding or EGFR activation resulted in cellular proliferation. Moreover, using a genetics-based approach, we found that the effects of AR signaling in MCF-10A cells were mediated through the cyclin-dependent kinase (CDK) inhibitor p21. These data further elucidate the mechanisms that affect AR signaling, and therefore may aid in the development of drugs targeting AR for breast cancer therapy.

## Materials and methods

### Ethics approval

This was a pre-clinical study not involving human subjects, and therefore did not require ethical review by an institutional review board.

### Plasmids and cell culture

AR cDNA was cloned into a modified version of the pIRESneo3 vector (Clontech, Mountain View, CA, USA), a bicistronic vector with an internal ribosomal entry site (IRES) and the gene encoding neomycin resistance (see Additional file 1 Table 1 for primers used). All cells [there seems to be some text deleted here from our manuscript. Should be along "All cells were purchased from ATCC"] (American Type Culture Collection (ATCC), Manassas, VA, USA) and grown at 37°C with 5% CO_2_. MCF-7, MDA-MB-231, and MDA-MB-453 cells were grown in DMEM (Invitrogen Corp., Carlsbad, CA, USA) supplemented with 5% FBS (Hyclone Laboratories Inc., Logan, UT, USA), 100 U/ml penicillin and 100 μg/ml streptomycin (both Invitrogen Corp.). Clones of MDA-MB-231 stably overexpressing AR cDNA were isolated and propagated in DMEM:F12 (1:1) without phenol red (Invitrogen Corp.), supplemented with 5% charcoal dextran-treated FBS (Hyclone Laboratories Inc.), 100 U/ml penicillin, 100 μg/ml streptomycin, and 500 μg/ml G418 (all Invitrogen Corp.). The non-transformed human breast epithelial cell line MCF-10A [35] was grown in DMEM:F12 (1:1) supplemented with 5% horse serum (GIbco, Carlsbad, CA, USA), 20 ng/ml epidermal growth factor (EGF), 10 μg/ml insulin, 0.5 μg/ml hydrocortisone, and 0.1 μg/ml cholera toxin (all Sigma-Aldrich, St. Louis, MO, USA) unless otherwise noted. All MCF-10A derivatives were selected on medium containing 120 μg/ml G418. Cells designated as Androgen Receptor In Breast Epithelium (ARIBE) cells were isolated and propagated in DMEM:F12 (1:1) without phenol red, supplemented with 5% charcoal dextran-treated FBS (Hyclone Laboratories Inc.). Two representative clones, ARIBE-1 and ARIBE-2, were used for all subsequent experiments.

Generation of the MCF-10A p21^-/- ^cell line has been described previously [36]. Because these cells utilize both neomycin and hygromycin for disruption of the *p21 *gene, AR cDNA was subcloned into pIRESpuro2 (Clontech). Cells were selected on medium containing 0.4 μg/ml puromycin (Sigma), and propagated in the same medium as used for the ARIBE cells.

### Cell-proliferation assays

For crystal violet staining, ARIBE and control cells were seeded in 25 cm^2 ^tissue-culture flasks at 10^5 ^cells/flask. The medium was changed 24 hours later and appropriate drug or vehicle added. All drugs and vehicle controls constituted 0.1% of the final volume in the flasks. The synthetic androgen R1881 (Perkin Elmer LAS, Boston, MA, USA) was diluted in ethanol (EtOH) and used at a concentration of 1 nmol/l. The androgen antagonist bicalutamide (Toronto Research Chemicals, Ontario, Canada) was diluted in methanol and used at a concentration of 10 nmol/l. Control cells were treated with EtOH only. After 3 days, or when the cells in the control flasks were at 90% to 95% confluency, all flasks were stained with crystal violet (Sigma) diluted in formalin to a concentration of 2 mg/ml.

All other growth assays were performed in 12-well tissue-culture plates. Cell counting was performed on a viability analyzer (Vi-Cell; Beckman Coulter, Fullerton, CA, USA), and all counts were performed in triplicate and repeated at least three times. For ARIBE cell assays, exponentially growing ARIBE and control cells were washed with Hank's balanced salt solution (HBSS; Invitrogen Corp.) three times, and seeded in DMEM:F12 medium without phenol red, using 2% charcoal dextran-treated serum, 10 μg/ml insulin, 0.5 μg/ml hydrocortisone, and 0.1 μg/ml cholera toxin, either without EGF or with 20 ng/ml EGF, as indicated. Cells were seeded at a density of 1.5 × 10^4 ^cells/well for experiments without EGF and at 5 × 10^3 ^cells/well for experiments using 20 ng/ml EGF. Medium was changed 24 hours later (day 0), and the drug or vehicle was added to the appropriate wells. All drugs and vehicle controls constituted 0.1% of the final volume in the wells. Medium was changed every other day until cells were counted. Cells were counted on days 0, 4, and 8 for experiments without EGF, and days 0, 2, and 4 for experiments using 20 ng/ml EGF.

MDA-MD-231 cell assays were performed similarly. Cells were seeded in DMEM:F12 (1:1) without phenol red, supplemented with 2% charcoal dextran-treated serum at a density of 10^4 ^cells/well. Cells were counted on day 4. The MAPK kinase (MEK) inhibitor U0126 (Promega Corp., Madison, WI, USA) [37] was diluted in DMSO (Sigma) and used at a concentration of 1 μmol/l.

### Immunoblotting and quantification

Whole-cell protein extracts prepared in Laemmli sample buffer were resolved by SDS-PAGE using 4% to 12% polyacrylamide gels (NuPage; Invitrogen Corp.), transferred to PVDF membranes (Invitrolon; Invitrogen Corp.), and probed with primary and horseradish peroxidase-conjugated secondary antibodies. Primary antibodies were rabbit polyclonal anti-p44/p42 MAPK (ERK) (9102; Cell Signaling Technology, Danvers, MA, USA), mouse monoclonal anti-phospho-p44/p42 MAPK (ERK) (Thr-202/Tyr-204) (9106; Cell Signaling Technology), mouse monoclonal anti-AR (sc-7305; Santa Cruz Biotechnology, Santa Cruz, CA, USA), mouse monoclonal anti-p21/WAF (#OP64; Calbiochem, Gibbstown, NJ, USA), rabbit polyclonal anti-glucocorticoid receptor (7437; Cell Signaling Technology) and mouse monoclonal anti-GAPDH (6C5) (ab#8245; Abcam, Cambridge, MA, USA). Proteins were visualized with chemiluminescence (Western Lightning Plus; Perkin Elmer). All blots were quantified using ImageJ software [38] and were normalized to their respective GAPDH loading control.

### Cell cycle analysis

Cells were seeded in six-well plates, and drug or vehicle was added the following day. Cells were then harvested and analyzed by fluorescence-activated cell sorting (FACS) analysis at 6 hours or 36 hours after drug addition. Cells were fixed in PBS with 3% formaldehyde and 0.4% NP-40, containing 2 μg/ml dye (Hoechst 33258; Invitrogen Corp.). DNA content was measured with a flow cytometer (BD LSR; BD Biosciences, San Jose, CA, USA), and percentages of G1/G0, S, and G2/M phase cells were determined using Modfit LT software (Verity Software House, Topsham, ME, USA).

### Luciferase assays

All luciferase experiments were performed in duplicate and repeated at least twice. Cells were seeded in 96-well plates (Microtest Optilux, BD Falcon, Franklin Lakes, NJ, USA) at 15% confluency in the medium described above for the cell-proliferation assays with 20 ng/ml EGF. At 24 hours after seeding, cells were transfected and concurrently treated with either 1 nmol/l R1881 or vehicle control. The transfection mixture per well contained 12.5 μL reduced serum medium (Opti-MEM; Invitrogen Corp.), 0.5 μL transfection reagent (Fugene 6; Roche USA, Nutley, NJ, USA), and 100 ng total plasmid. The reporter plasmids consisted of either three copies of a wild-type consensus binding site for AR, or three copies of a mutated binding site of AR conjugated to a firefly luciferase reporter. The consensus binding sites used in the construction of the reporters were gtacattGtGttct for AR, and gtaAattGtAttTt for the mutated AR consensus binding sites. Additionally, each well was co-transfected with a *Renilla *luciferase plasmid to serve as control for transfection efficiency.

At 48 hours after drug treatment and transfection, luciferase activity was assayed using a commercial luciferase assay system (Dual-Glo; Promega Corp.) following the manufacturer's protocol. Briefly, cells were lysed with 150 μL of a 1:1 mixture of luciferase (Dual-Glo) and medium (Opti-MEM), and incubated at room temperature for 15 minutes. Firefly luciferase activity was measured by a luminometer (TopCount NXT; Perkin Elmer) for 2 seconds per well, then quenched with 75 μL of the provided reagent (Stop & Glo; Promega Corp.). Fifteen minutes after quenching firefly luciferase activity, *Renilla *luciferase activity was measured on the luminometer for 2 seconds per well. All firefly luciferase measurements were normalized to *Renilla *luciferase measurements. AR activity was expressed as the ratio of the luciferase activity in cells transfected with consensus binding site plasmids divided by the luciferase activity in cells transfected with mutated binding site plasmids.

### cDNA synthesis and quantitative real-time reverse transcriptase PCR

Total RNA was extracted from cells (RNeasy Mini Kit; Qiagen Inc., Valencia, CA, USA) according to the manufacturer's instructions, with on-column DNAse I digestion, then complementary DNA was synthesized (First Strand cDNA Synthesis Kit; GE Amersham, Pittsburgh, PA, USA). Using cDNA as template and SYBR Green (Invitrogen Corp.) to detect DNA products, expression of insulin-like growth factor (IGFR)-1 was assayed using a two-color real-time detection system (MyiQ; (BioRad, Hercules, CA, USA). Reactions were performed in triplicate, and repeated at least twice. Transcript levels were normalized to TATA binding protein (*TBP*) levels in the same samples in each experiment. *FKBP5, NSDHL *and *IGFR-1 *gene expression was reported as a ratio of the expression in cells treated with drug divided by the expression in cells treated with vehicle. Quantitative real-time reverse transcription PCR (qPCR) was performed (for primers, see Additional file 1 Table 1).

### Fluorescent *in situ *hybridization analyses

For fluorescent *in situ *hybridization (FISH) analysis, tissue pre-treatment was first performed using paraffin wax-embedded tissue sections 4 to 5 μm thick that had been mounted on charged microscope slides, which were dewaxed, rehydrated through a decreasing graded ethanol series, and treated using a commercial tissue protease kit (Pre-treatment Kit III; Abbott Molecular Inc.,/Vysis Inc., Downers Grove, IL, USA) according to the manufacturer's directions. After halting protease activity with the provided stop solution, slides were washed, then dehydrated through an increasing graded ethanol series and taken to hybridization. Fluorescently labeled hybridization probes for the X centromere (CEP X Alpha SpectrumGreen; Abbott Molecular/Vysis) and the AR locus at Xq12 (AR gene probe SpectrumOrange; Abbott Molecular/Vysis) were diluted in hybridization buffer (50% formamide, 2 × saline sodium citrate (SSC), 10% Dextran Sulfate), mounted on slides, covered with coverslips, and denatured at 95°C for 5 minutes. Hybridization was conducted overnight at 37°C. Slides were then washed for 4 minutes at room temperature in post-hybridization wash buffer (2 × SSC, 0.3% NP-40), followed by a second wash with SSC at 75°C for 3 minutes, and then a third wash with water at room temperature for 4 minutes. Slides were then counterstained with DAPI (1:10,000 dilution in water from 5 mg/ml stock; Sigma Chemicals) for 5 minutes at room temperature. Coverslips were mounted using anti-fade mounting medium (Prolong Gold[ Invitrogen Corp./Molecular Probes), and the slides were sent for analysis. Slides were imaged with an epifluorescence microscope (50i; Nikon, Tokyo, Japan) equipped with an illuminator (X-Cite series 120; EXFO Photonics Solutions Inc., Ontario, CA, USA) and a 10 ×/1.4 NA oil immersion lens (Neofluar; Carl Zeiss Inc., Thornwood, NY, USA). Fluorescence excitation/emission filters were as follows: SpectrumOrange excitation, 546 nm/10 nm BP; emission, 578 nm LP (Carl Zeiss Inc.); DAPI excitation, 330 nm; emission, 400 nm via an XF02 fluorescence set (Omega Optical, Brattleboro, VT, USA); SpectrumGreen excitation, 475 nm; emission, 535 nm via a combination of 475RDF40 and 535RDF45 filters (Omega Optical). Grayscale images were captured for presentation using NIS-Elements software (Nikon) and an attached digital camera (CoolsnapEZ; Photometrics, Tucson, AZ, USA), pseudo-colored, and merged.

### Tissue Microarrays

A previously described (@) breast cancer tissue microarray was used for FISH analysis. Two blocks were employed consisting of 30 and 35 (total = 65) samples of primary invasive ductal carcinomas. Tissue microarrays were prepared for FISH analysis as described above.

### Enzyme linked immunosorbent assays (ELISA)

For PSA ELISAs, growth assays were performed as above and supernatants harvested at the end of Day 4. Supernatants were then subjected to ELISAs using the Quantikine human Kallekrein3/PSA Immunoassay kit (R & D systems, Minneapolis, MN) as per the manufacturer's protocol.

### Statistical analysis

All statistical analyses were performed using GraphPad InStat software (La Jolla, CA, USA). *P *< 0.05 was considered significant.

## Results

### Androgen receptor is not amplified in human breast cancers

As mentioned above, many previous studies have identified AR expression in human breast cancers. However, levels of AR expression (that is, AR overexpression), have been infrequently reported due to difficulty with quantification by immunohistochemistry staining. However, recent studies suggest that overexpression of AR in breast cancer does occur, and is associated with overexpression of ERα and in breast cancers with *PIK3CA *mutations in the kinase domain [24,39]. In addition, AR overexpression and AR gene amplification have been reported in prostate cancers [40]. Although ERα gene amplification in breast cancers is controversial [41], we performed FISH analysis on tissue microarrays (TMAs) with known AR-positive breast cancers using a gene probe for AR and a centromeric chromosome X probe to query for AR gene amplification. There were approximately two copies of AR for every two copies of chromosome X in primary breast cancer samples. Although overexpression is difficult to quantify, the complete lack of AR gene amplification strongly suggests that gene amplification is not a common event in human breast cancers. The cell line E006AA has a known AR amplification [42] and was used as a positive control for this assay (see Additional file 1 supplementary Figure 1). Similar to ERα, the results confirm that in the high percentage of breast cancers that express AR, gene amplification does not seem to be a major underlying genetic change.

**Figure 1:**
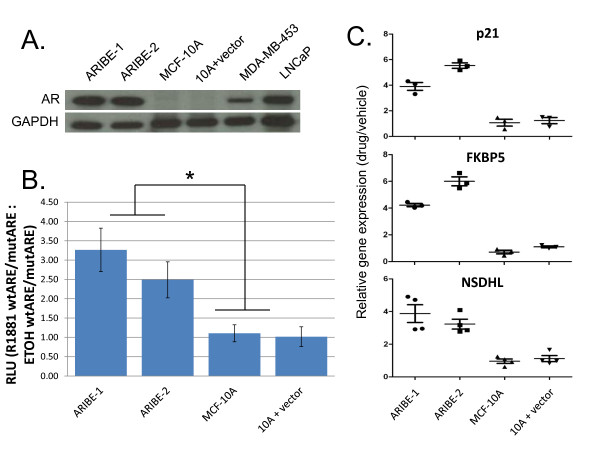
**Expression of androgen receptor (AR) in human breast epithelial cells**. **(A) **MCF-10A cells were stably transfected with an AR cDNA, and lysates from single-cell clones were probed for AR expression by western blotting. As a negative control, MCF-10A cells were also transfected with empty vector (10A plus vector) and isolated as single-cell clones. MDA-MB-453 and LNCaP cell lysates served as positive controls for AR expression. All lysates were also probed with GAPDH antibody as a loading control. **(B) **Androgen Receptor In Breast Epithelium (ARIBE) cells and control cells were transfected with luciferase reporter plasmids containing consensus DNA binding sites and mutated controls for AR as described in Methods. At the time of transfection, cells were treated with 1 nmol/l R1881 or vehicle (ethanol), and analyzed 48 hours later. Firefly luciferase measurements were normalized to *Renilla *luciferase measurements in all samples. The relative luciferase units (RLU) ratio was calculated as the luciferase expression comparing wild-type ARE/mutant ARE in R1881 versus vehicle-treated control cells (R1881 wtARE/mutARE: ETOH wtARE/mutARE). Error bars represent the standard deviation of three independent experiments. Luciferase expression in each ARIBE clone compared with control cell lines was significant by one-way analysis of variance (ANOVA) (*P *< 0.05).**(C) **cDNA was made from RNA of cells treated with 1 nmo/l R1881 or vehicle for 48 hours. Quantitative real-time PCR using SYBR Green was performed on triplicate samples of each cell line using intron-spanning primers for each of three androgen-response genes. All cycle threshold numbers were normalized to a control gene, TATA binding protein (TBP). Ratio is expression in cells treated with drug versus vehicle. Error bars represent the standard deviation of four independent experiments. In both ARIBE lines, induction of all genes after drug treatment was significant by one-way ANOVA compared with control cell lines (*P *< 0.001).

### Stable expression of androgen receptor in human breast cells

To study AR signaling in ERα-negative non-tumorigenic human breast epithelial cells, we transfected MCF-10A cells with an AR cDNA using a bicistronic vector with an IRES and the gene encoding neomycin resistance. Multiple clones were isolated and designated as ARIBE cells with two representative clones, ARIBE-1 and ARIBE-2, used for all subsequent experiments. As a control, MCF-10A cells were transfected with an 'empty' vector and underwent the same antibiotic selection and single-cell dilution process. Western blot analysis identified high levels of expression of AR in ARIBE-1 and ARIBE-2, which was higher than the expression in MDA-MB-453 cells, but comparable with levels in the AR-positive prostate cancer cell line LNCaP (Figure 1A; see Additional file 1 supplementary Table 2). As expected, MCF-10A parental cells and the MCF-10A empty vector control had no appreciable AR expression.

We initially characterized the effects of AR ligand binding on ARIBE cells using a luciferase reporter system, and examined changes in AR response genes using qPCR. The luciferase reporter system employs plasmids that contain a firefly luciferase reporter gene driven by either a wild-type consensus binding site for AR (androgen-response element; ARE) or a mutated ARE that has been shown to have reduced binding affinity for AR. If AR is active, it will drive luciferase expression when transfected with the wild-type plasmid but not with the mutant plasmid. In all experiments, a *Renilla *luciferase plasmid was co-transfected with the firefly luciferase plasmid as a control for transfection efficiency. We assayed the activity of AR in our ARIBE cell lines and in control cell lines cultured with the synthetic androgen R1881 or vehicle control. R1881 is a non-aromatizable synthetic analog of testosterone, and has been shown to saturate AR binding sites in certain breast cancer cell lines at concentrations in the range of 1 to 100 nmol/l [30]. The relative ratio of luciferase activity of the wild-type ARE to mutant ARE was significantly increased in R1881-stimulated conditions relative to treatment with vehicle only (EtOH) (that is, R1881 wtARE/mutARE: ETOH wtARE/mutARE) in the two ARIBE clones compared with the control cell lines (Figure 1B). To show that AR stimulated by ligand in ARIBE cells also affected gene expression of endogenous AREs, we performed qPCR on known AR response genes. Prostate-specific antigen (PSA) is the prototypical AR response gene, and has been reported to be expressed and secreted by some breast cancer cell lines, although many AR-positive breast cancer cell lines do not produce PSA upon AR ligand binding [43]. Similarly, we did not detect PSA in ARIBE cell cultures either by qPCR of cellular mRNA or by ELISA of cell supernatant, although we could readily detect PSA from the prostate cancer cell line LNCaP upon R1881 stimulation (data not shown). Because of the inability to use PSA as a marker for AR signaling, we examined other known androgen-responsive genes including *IGFR-1 *[44,45], *p21 *[46], *FKBP5 *[47] and *NSDHL *[48]. qPCR was performed on mRNA derived from ARIBE cells and controls to determine the change in gene expression of these four genes when stimulated with AR ligand. After 24 and 48 hours of AR ligand exposure, there was significantly increased induction of *p21, FKBP5 *and *NSDHL *expression in ARIBE cells compared with MCF-10A or vector control cell lines when stimulated with R1881 (data for 48 hours shown in Figure 1C). IGFR-1 expression was significantly induced at 24 hours (see Additional file 1 supplementary Figure 2) after AR ligand exposure, but was not significantly upregulated at the 48-hour time point relative to controls.

**Figure 2:**
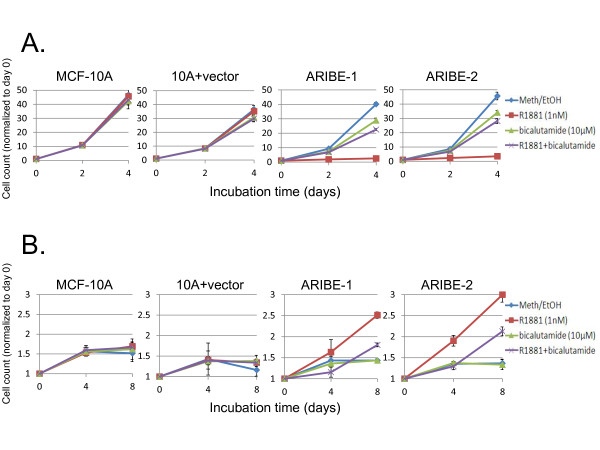
**Activation of the mitogen-activated protein kinase (MAPK) pathway alters the growth response of Androgen Receptor In Breast Epithelium (ARIBE) cells to androgen receptor (AR) ligand**. **(A) **Control cells (MCF-10A and 10A plus vector) and two ARIBE clones were cultured in normal propagation media (with 20 ng/ml epidermal growth factor (EGF)) and treated with vehicle, 10 μmol/l bicalutamide, 1 nmol/l R1881, or a combination of R1881 and bicalutamide. Cells were counted after either 2 or 4 days of treatment, and normalized to values of cells counted on the day of drug addition (day 0). Error bars represent the standard deviation of the mean of three independent cell counts. The growth difference between cells treated with R1881 and R1881 plus bicalutamide was significant by Student's two-tailed *t*-test (*P *< 0.001). **(B) **Cells were cultured under normal propagation conditions except for the absence of EGF from the medium. Cells were treated with vehicle or drugs as above. Cells were counted after either 4 or 8 days of treatment, and normalized to values of cells counted on the day of drug addition (day 0). Error bars represent the standard deviation of the mean of three independent cell counts. The growth difference between cells treated with R1881 and R1881 plus bicalutamide was significant by Student's two-tailed *t*-test (*P *< 0.01).

### Proliferative response to androgen receptor ligand in Androgen Receptor In Breast Epithelium cells

Because the growth response to AR ligands in breast cells can vary depending on the cell line, we next evaluated any proliferative effects of R1881 on ARIBE cells. Treating ARIBE cells with 1 nmol/l R1881 resulted in significant (*P *< 0.001) growth inhibition (Figure 2A; see Additional file 1 supplementary Figure 3A). To confirm that this effect was due to signaling through AR, we concurrently treated the cells with the androgen antagonist bicalutamide. When bicalutamide was used in combination with R1881, the inhibitory effect of R1881 was greatly diminished, restoring cell proliferation to levels close to those seen with bicalutamide alone or vehicle control (Figure 2A; see Additional file 1 supplementary Figure 3A). In addition, ARIBE cells showed a dose-dependent inhibitory response to serial dilutions of R1881 (see Additional file 1 supplementary Figure 3B). The observed half-maximal inhibitory concentration (IC50) was approximately 60 pmol/l which is consistent with that from reports of other cell lines [49-51] implying that our model is accurately recapitulating AR signaling. To determine optimal phenotypic changes as a result of AR signaling, we performed a time-course analysis of ARIBE cells exposed to AR ligand (see Additional file 1 supplementary Figure 4). A marked difference in growth was seen at 48 hours, and the difference between ARIBE cells treated with R1881 versus vehicle continued to increase with prolonged exposure to these culture conditions. Based on these results, a 48-hour exposure to R1881 was used for assessing downstream AR signaling events for subsequent experiments.

**Figure 3:**
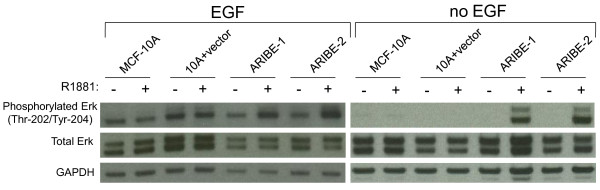
**Androgen Receptor In Breast Epithelium (ARIBE) cells stimulate mitogen-activated protein kinase (MAPK) signaling upon androgen receptor (AR) ligand binding**. Control cells (MCF-10A and 10A plus vector) and ARIBE cells were cultured under normal propagation conditions in the presence or absence of 20 ng/ml epidermal growth factor (EGF), treated with either vehicle or 1 nmol/l R1881 for 48 hours, and then used to make lysates for western blotting as described in Methods. Blots were probed for phosphorylated extracellular signal-regulated kinase (ERK) (Thr-202/Tyr-204) and total ERK. GAPDH antibody was used as loading control.

**Figure 4:**
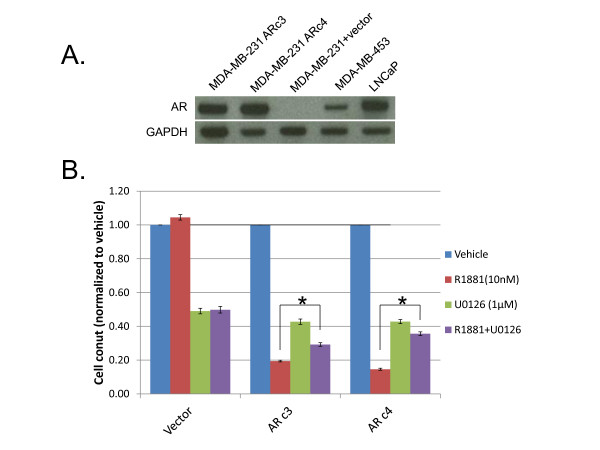
**MDA-MB-231 cells transfected with androgen receptor (AR) show growth inhibition when treated with androgen**. **(A) **The breast cancer cell line MDA-MB-231 was transfected with an AR cDNA, and lysates from single-cell clones were analyzed for AR protein expression. Two representative clones are shown. MDA-MB-453 and LNCaP lysates served as positive controls for AR expression. Lysates were also probed with GAPDH as a loading control.**(B) **Two MDA-MB-231 clones transfected with AR (ARc3 and ARc4) and control cells (Vector) were treated with vehicle, 1 μmol/l of the MEK inhibitor U0126, 1 nmol/l of R1881, or a combination of R1881 and U0126. Cells were counted after 4 days of drug treatment, and normalized to counts of vehicle-treated control cells. Error bars represent the standard deviation of the mean of three independent counts. The growth difference between cells treated with R1881 and R1881 plus U0126 was significant by Student's two-tailed *t*-test (*P < 0.005).

Previous studies in our laboratory have shown that manipulation of mitogenic factors can influence the response to nuclear hormone receptor ligands [34]. MCF-10A cells provide an ideal system to study these effects because normal propagation requires EGF, and removal of this growth factor results in inactivation of MAPK signaling and a complete arrest of cell cycle in G1[34,35,52]. Interestingly, removal of EGF from cell cultures reversed the effects of R1881, resulting in proliferation rather than growth inhibition of ARIBE cells, which was significant (*P *< 0.01) (Figure 2B). Without EGF in the culture medium, the doubling time of ARIBE cells treated with R1881 was much longer than the doubling time of MCF-10A or ARIBE cells cultured in medium with EGF and no R1881 (Figure 2A versus Figure 2B). Therefore, the cell-proliferation assay for ARIBE cells cultured in R1881 without EGF was carried out for 8 rather than 4 days. Nonetheless, the effects were obvious and highly reproducible. Moreover, the addition of bicalutamide antagonized the effect of R1881 in ARIBE cells in both EGF-containing and EGF-free conditions, indicating that both growth inhibition and cell proliferation were mediated through AR signaling.

### Effects of androgen receptor signaling in Androgen Receptor In Breast Epithelium cells

To determine whether the growth inhibition induced by R1881 was the result of cell death or cell cycle arrest, we performed FACS analyses on ARIBE cells cultured in the presence of EGF with and without R1881. There was no significant difference between vehicle-treated and drug-treated cells at 6 hours, but at 36 hours, the cells treated with R1881 showed an increase in the G1/G0 cell cycle fraction compared with cells treated with vehicle (see Additional file 1 supplementary Figure 5A). Cells treated with R1881 arrested in G1/G0 but remained viable, as shown by the fact that replacement of the culture medium with medium containing EGF but without R1881 restored a normal cell cycle profile within 48 hours (see Additional file 1 supplementary Figure 5B).

**Figure 5:**
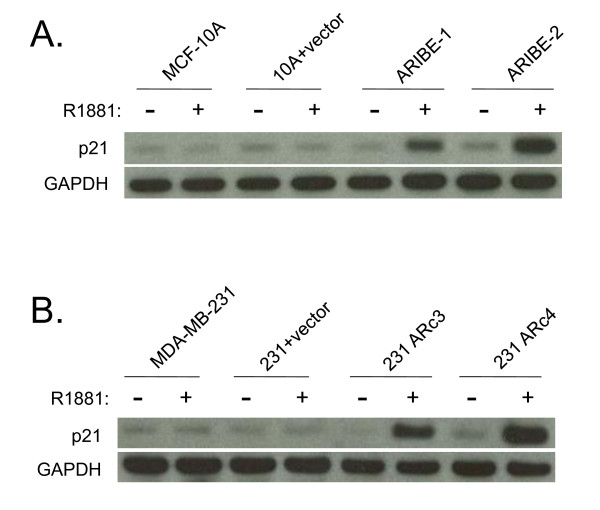
**Upregulation of p21 expression upon androgen receptor (AR) ligand binding in the presence of mitogen-activated protein kinase (MAPK)signaling**. **(A) **Control cells (MCF-10A and 10A plus vector) and ARIBE cells were cultured in normal propagation media (with 20 ng/ml EGF) and treated with either vehicle or 1 nmol/l R1881 for 24 hours. Whole-cell lysates were probed for expression of p21 and GAPDH for a loading control. **(B) **Control cells (MDA-MB-231 and 231 plus vector) and MDA-MB-231 cells expressing AR (ARc3 and ARc4) were cultured in the presence EGF and treated with either vehicle or 1 nmol/l R1881 for 24 hours. Whole-cell lysates were probed for expression of p21 and GAPDH was used as loading control.

We next determined the signaling pathways that were activated by AR-mediated cell proliferation in ARIBE cells. Previous studies have reported that AR signaling can activate the MAPK pathway via phosphorylation of extracellular signal-regulated kinase (ERK) [53]. We examined phospho-ERK levels in our ARIBE cells under R1881-induced proliferative conditions. Cells were seeded in medium without EGF, and exposed to R1881 or vehicle control for 48 hours, then harvested for cell lysates. As expected, control cell lines had no appreciable increase in phosphorylated ERK levels whereas ARIBE cells had a marked increase in phosphorylated ERK when treated with R1881 (Figure 3, right panel; see Additional file 1 supplementary Table 2). These data are consistent with previous reports that AR signaling can lead to a mitogenic response via MAPK activation, and lend further support to the notion that ARIBE cells demonstrate physiologic AR signaling. Interestingly and seemingly paradoxically, the growth-inhibitory phenotype seen with the full dose (20 ng/ml) of EGF also showed increased phosphorylation of ERK in ARIBE cells treated with R1881 (Figure 3, left panel; supplementary Table 2) suggesting that the growth-inhibitory response may be due to overactive MAPK signaling (see Discussion).

Collectively, these data suggest that ARIBE cells exposed to R1881 display physiologic AR signaling, based upon cellular growth patterns that are antagonized by bicalutamide, activation of key signal transduction pathways, and the ability to upregulate gene expression via known AREs.

### Androgen receptor signaling in breast cancer cells

To ensure that the results seen with ARIBE cells were due to signaling through AR and were not a unique response of MCF-10A cells or artifacts from random transgene insertion, we created a second AR-expressing cell line. We used the MDA-MB-231 cell line because it is also ERα/PR/HER2-negative, and has a defined number of mutations in key oncogenes (Sanger COSMIC database). This cell line overexpresses EGFR, which leads to autophosphorylation of EGFR and constitutive activation of the MAPK pathway [54,55]. MDA-MB-231 cells also harbor a *KRAS *mutation and a *BRAF *mutation, both of which could further activate the MAPK pathway. However, it has been shown that this cell line is relatively genetically stable compared with other breast cancer cell lines [23]. We subjected he MDA-MB-231 cells to the same protocol performed on MCF-10A cells, and western blot analysis of the 231 plus AR clones found similar levels of AR expression to those found in MCF-10A cells (Figure 4A; see Additional file 1 supplementary Table 2).

A control cell line was also created by transfecting MDA-MB-231 cells with the empty vector and selecting antibiotic-resistant clones. When stably expressing AR, these cells showed similar responses to R1881 as seen in ARIBE cells; that is, growth inhibition occurred in a dose-dependent manner but with a higher IC50 compared with ARIBE cells, and this effect was blocked by co-culture with bicalutamide (data not shown). A potential caveat to these studies is that R1881 has been shown to bind to the glucocorticoid receptor (GR) [56], and therefore expression of GR was examined in all cell lines. We found that cell lines with AR expression did indeed express GR, but GR expression was also seen in the parental cell lines and in empty vector control cell lines that do not express AR (see Additional file 1 supplementary Figure 6 and Supplementary Table 2). The fact that GR expression was present in all cell lines, in conjunction with the demonstration that the AR antagonist bicalutamide blocked the effects of R1881 only in AR-expressing clones, strongly supports that our model systems accurately reflect physiologic AR signaling.

**Figure 6:**
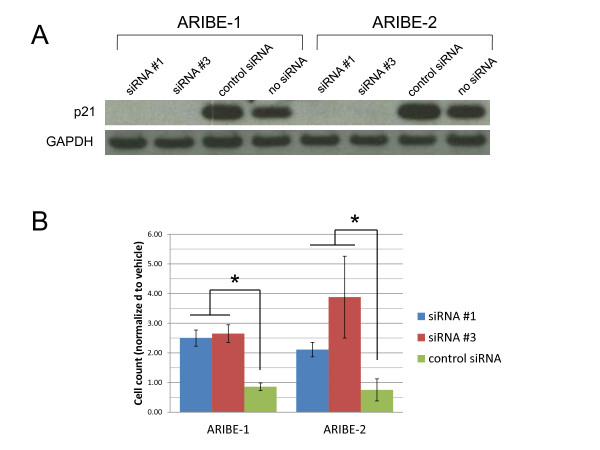
**p21 knock-down via siRNA abrogates growth inhibition of Androgen Receptor In Breast Epithelium (ARIBE) cells**. **(A) **ARIBE cells were transfected with p21 siRNA constructs, control siRNA, or no siRNA, and incubated for 24 hours. After incubation, whole-cell lysates were probed for expression of p21, and GAPDH was used as a loading control. **(B) **ARIBE cells transfected for 24 hours with p21 or control siRNA were subsequently treated with vehicle or 1 nmol/l R1881 for 4 days, and cells were counted. The ratio of growth between vehicle and R1881-treated cells was calculated and normalized to untransfected (no siRNA) values to determine the fold increase in growth of siRNA-transfected cells. Error bars represent the standard deviation of the mean of three independent counts. The growth difference between cells transfected with p21 siRNA constructs and mock siRNA was significant by one-way analysis of variance (*P < 0.05).

Because of the aforementioned genetic alterations in the MAPK pathway in MDA-MB-231 cells, no exogenous growth factors are needed for propagation. Therefore, to simulate EGF removal, we used pharmacological inhibitors of the MAPK pathway and then assayed the response to R1881. We used the MEK inhibitor U0126 [37] because this inhibitor would theoretically be active in cells with *RAS *and *RAF *mutations, given that MEK is distal to these proteins in the MAPK pathway. R1881 had no effect on empty vector control cells but caused marked growth inhibition in two AR-expressing clones (Figure 4B). Addition of 1 μmol/l U0126 produced significant toxicity in all three cell lines regardless of AR expression, but also produced the expected effect of reversing the response to R1881 in AR-expressing clones (*P *< 0.005). Collectively, these results and the ARIBE cell line data show that AR signaling with concurrent MAPK activation via the EGFR pathway can lead to cell cycle arrest. Additionally, these observations led us to hypothesize that the cells are undergoing a phenomenon similar to oncogene-induced senescence, whereby the hyperstimulation of growth-promoting pathways and/or DNA damage may induce cellular death/arrest or induce senescence [57].

### Androgen receptor signaling is mediated by p21 in breast epithelial cells

The CDK inhibitor p21 is involved in regulating cell cycle progression, specifically in mediating G1 arrest [58]. We found that under conditions of EGF stimulation, ARIBE cells treated with R1881 underwent arrest in the G1/G0 phase of the cell cycle (see Additional file 1 supplementary Figure 5) and that *p21 *gene expression increased in ARIBE cells in response to R1881 (Figure 1C).

We further examined p21 expression in ARIBE cells treated with R1881, using western blotting. Within 24 hours of stimulation with AR ligand, ARIBE cells displayed upregulation of p21 protein expression (Figure 5A; see Additional file 1 supplementary Table 2). A similar result was seen in the MDA-MB-231 breast cancer cell lines that overexpress AR (Figure 5B; see Additional file 1 supplementary Table 2). It has been well described that cell cycle arrest mediated by p21 occurs via its induction by p53 [59]. However, in ARIBE cells induction of p21 appeared to be independent of p53 function, as the increased p21 levels induced by R1881 did not correlate with increased levels of p53 protein (data not shown).

Additionally, we examined the expression of cyclin E and cyclin D1, two key regulators of cell cycle progression [60] After 48 hours of treatment with R1881 in the presence of EGF, cyclin E levels were not altered in ARIBE or control cell lines (data not shown), but levels of cyclin D1 were decreased by nearly 50% compared with control cell lines (see Additional file 1 supplementary Figure 7 and supplementary Table 2).

**Figure 7:**
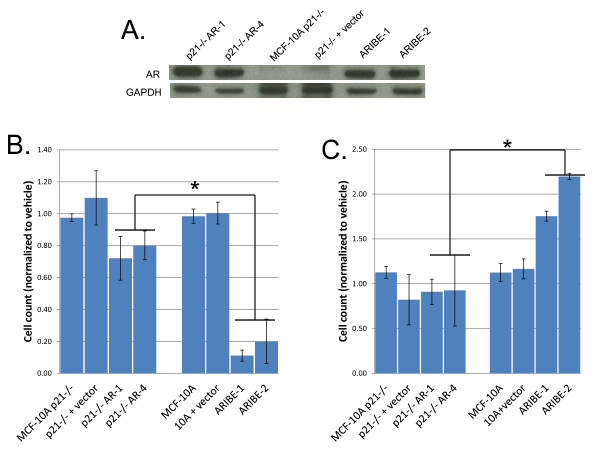
**p21 mediates growth effects of androgen receptor (AR) ligand in MCF-10A cells**. **(A) **MCF-10A p21^-/- ^cells were stably transfected with an AR cDNA, and lysates from single-cell clones were probed for AR expression by western blotting. As a negative control, MCF-10A p21^-/- ^cells were also transfected with empty vector (p21^-/- ^plus vector) and isolated as single-cell clones. Expression of AR was compared with ARIBE cells, which have wild-type p21. MDA-MB-453 and LNCaP cell lysates served as positive controls for AR expression. All lysates were also probed with GAPDH antibody as a loading control. **(B) **The p21^-/- ^(left) and p21 wild-type (right) cells were cultured in normal propagation media with 20 ng/ml epidermal growth factor (EGF), and treated with 1 nmol/l R1881. Cells were counted after 4 days of treatment, and normalized to values of cells counted on the day of drug addition (day 0). Error bars represent the standard deviation of the mean of three independent cell counts. The growth difference between p21^-/- ^AR-expressing cells and p21-wild-type AR-expressing cells was significant by one-way analysis of variance (ANOVA) (*P < 0.001). **(C) **Cells were cultured under normal propagation conditions except for the absence of EGF from the medium. Cells were treated with vehicle or drugs as described above. Cells were counted after 8 days of treatment, and normalized to values of cells counted on the day of drug addition (day 0). Error bars represent the standard deviation of the mean of three independent cell counts. The growth difference between p21^-/- ^AR-expressing cells and p21-wild-type AR-expressing cells was significant by one-way ANOVA (*P < 0.01).

Taken together, these results suggest that dual exposure to EGF and R1881 leads to reduced cellular proliferation, as evidenced by increases in the CDK inhibitor p21 and decreases in cyclin D1 protein levels.

Although the striking increase in p21 protein levels upon R1881 exposure and the known role of p21 in mediating cell cycle arrest suggested that p21 might be a key mediator of AR-induced arrest, we sought to definitively confirm this using two complementary techniques: knock-down of *p21 *gene expression via RNA interference (RNAi), and somatic cell gene knock-out, as described previously [61]. After transient transfection of siRNA into ARIBE cells a dramatic reduction in p21 protein was seen, compared with cells transfected with either a 'scrambled' control siRNA or with no siRNA (Figure 6A; see Additional file 1 supplementary Table 2). Transfected cells were then examined by proliferation assays. For control transfected cells, R1881 treatment produced significant growth inhibition in the presence of EGF as expected (Figure 6B). However, in transfected cells with *p21 *gene knock-down, the ability of R1881 to cause cell cycle arrest under full EGF conditions (20 ng/ml) was dramatically reduced compared with control cells (*P *< 0.05). Because of the transient nature of siRNA and the longevity of the cell-proliferation assays in conditions with no EGF, effects of p21 knock-down on increased cell proliferation mediated by AR signaling could not be assessed.

Owing to this inability to assess the effect of *p21 *gene knock-down under conditions of increased cell proliferation (that is, without EGF), and the fact that gene knock-down can produce non-specific toxicity and significant biologic differences compared with gene knock-out [61], we next made use of our previously described MCF-10A somatic cell gene-targeted p21 null clones [36]. MCF-10A p21^-/- ^cells were stably transfected with the same AR cDNA used to create the ARIBE cell line. Clones with antibiotic resistance underwent a single-cell dilution process and multiple clones were isolated. Expression of AR was assayed by western blotting. Two representative clones (p21^-/- ^AR-1 and p21^-/- ^AR-4) had levels of AR expression comparable with those of the p21 wild-type ARIBE cells (Figure 7A; see Additional file 1 supplementary Table 2).

To determine if p21 knock-out recapitulated the RNAi experiments, ARIBE clones and p21^-/- ^AR cells were treated with R1881 in the presence of EGF. As shown previously, R1881 inhibited the growth of ARIBE cells. However, in cells with no functional p21, the effect of R1881 was greatly attenuated, as p21^-/- ^AR-1 and p21^-/- ^AR-4 clones did not show significant growth inhibition when treated with this AR ligand compared with p21 wild-type ARIBE cells (Figure 7B), similar to our p21 siRNA experiments. Predictably, bicalutamide did not have any effect in p21 null cells (see Additional file 1 supplementary Figure 8A).

**Figure 8:**
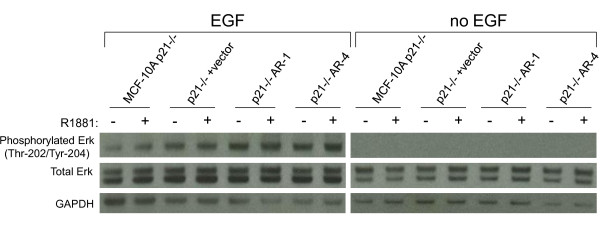
**p21^-/- ^cells do not stimulate mitogen-activated protein kinase (MAPK) signaling upon androgen receptor (AR) ligand binding**. Control cells (MCF-10A p21^-/- ^and p21^-/- ^plus vector) and AR-expressing cells (p21-/- AR-1 and p21^-/- ^AR-4) were cultured under normal propagation conditions in the presence or absence of 20 ng/ml epidermal growth factor (EGF), and treated with either vehicle or 1 nmol/l R1881 for 48 hours. Western blotting was performed on whole-cell lysates. Blots were probed for phosphorylated extracellular signal-regulated kinase (ERK) (Thr-202/Tyr-204) and total ERK. GAPDH antibody was used as a loading control.

We then tested ARIBE p21 wild-type and AR-positive p21 null cells with R1881 under conditions with no EGF. Somewhat unexpectedly, when cells were arrested via removal of EGF, p21^-/- ^AR cells did not show a growth-stimulated phenotype when treated with R1881, whereas the p21-wild-type ARIBE cells displayed the expected cell proliferation (Figure 7C). Consistent with this finding, bicalutamide did not affect responses to R1881 in p21 null cells under culture conditions with no EGF (see Additional file 1 supplementary Figure 8B). This could reflect the known paradoxical role of p21 in initiating cell cycle progression in arrested cells [62]. An alternative, but not mutually exclusive possibility is that if p21 is necessary for AR-induced MAPK signaling, then lack of p21 might prevent activation of this pathway and therefore nullify the growth-promoting effects of AR signaling in the absence of EGF stimulation. Indeed, it has been previously reported that cyclin/CDK complexes can affect the MAPK cascade [63]. Therefore we hypothesized that without functional p21, AR-expressing cells would not show any increase in MAPK signaling, which could explain the lack of effect seen under both full EGF and no EGF culture conditions.

To formally address this hypothesis, we repeated the experiments performed on ARIBE cells and examined the levels of phosphorylated ERK in AR-expressing p21 null cells. We found that exposure to R1881 was no longer capable of increasing levels of phosphorylated ERK in p21 null cells regardless of AR expression or EGF growth conditions (Figure 8; see Additional file 1 supplementary Table 2).

Together, these data strongly suggest that in human breast epithelial cells, AR signaling requires p21 for MAPK activation, and that the level of MAPK activation via EGFR and AR signaling ultimately determines the response of cellular proliferation versus cell cycle arrest.

## Discussion

Hormonal therapy is very successful for the treatment of breast cancer but remains limited to targeting the ERα pathway, as evidenced by the development of AIs and selective estrogen receptor modulators. However, drug resistance leading to recurrence of many of these ERα-positive breast cancers necessitates continuing efforts to develop new therapies. This has recently spurred interest in AR as a potential breast cancer target for treating ERα-positive hormone-resistant breast cancers. Moreover, 10% to 20% of ERα/PR-negative breast cancers are AR-positive, which potentially opens the possibility of hormone therapies for these breast cancers as well. Furthermore, the history of success in targeting nuclear receptors for cancer treatment (breast cancer, prostate cancer, acute promyelocytic leukemia) gives confidence that targeting AR for breast cancer therapy could be of tremendous importance in treating this disease, and indeed clinical trials are currently underway to test this hypothesis = [15]. Historically, side-effect profiles have limited the use of targeted AR therapies for breast cancer [21], but a more vexing problem has been the inability to predict response in pre-clinical models. Because AR ligands can have opposing and paradoxical effects in various breast cancer cell lines expressing AR, applying AR-targeted therapies for breast cancer treatment has been challenging. In an effort to understand the effects of AR signaling in breast tissues with the goal of exploiting this knowledge for therapy, we generated cellular models of AR expression using the ERα/PR-negative breast epithelial cell lines MCF-10A and MDA-MB-231. The MCF-10A cell line in particular has many advantages over the use of cancerous cell lines because it is genetically stable, it does not contain mutations in genes commonly mutated in breast cancer, and overexpression of nuclear hormone receptors results in physiologic signaling [34].

We characterized these cell lines using a variety of methods, and found that physiologic AR signaling is present in these cells and can induce increased transcription of genes via AREs and increased MAPK signaling. Importantly, our studies provide a number of mechanistic insights. First, R1881 bound to AR leads to increased MAPK signaling regardless of the growth phenotype. Second, AR signaling is dependent on the CDK inhibitor p21, as gene knock-down and knock-out largely abrogated all AR-mediated proliferation in these cell lines. Third, hyperactivation of the MAPK pathway by both EGFR and AR signaling leads to cell cycle arrest, whereas stimulation by either EGFR or AR alone results in cellular proliferation. Cellular arrest by EGFR and AR signaling may be similar to the phenomenon of oncogene-induced senescence, whereby activation of growth-promoting pathways beyond a critical threshold induces cell cycle arrest followed by senescence. Importantly, because our unique model is capable of displaying both growth phenotypes within the same cell line, it allows for the further study of genetic effectors that specifically mediate a growth stimulatory versus inhibitory response to AR signaling in human breast cells.

The fact that p21 is necessary for AR signaling leading to MAPK activation is consistent with previous reports that the p21 promoter contains an ARE [46]. Furthermore, our analyses showed that in both non-cancerous and cancerous human breast epithelial cells, AR ligand binding was associated with an increase in *p21 *gene expression regardless of the growth phenotype. This may have important clinical considerations, as we have previously reported that loss of p21 expression is seen in up to 40% of human breast cancers [36]. It might therefore be possible for p21 to be used as a negative predictive marker of response in AR-positive breast cancers that are otherwise eligible for future AR-targeted therapies. In addition, our results open the possibility of using activating genetic alterations or mutations of the MAPK pathway as potential positive predictors of response to AR-directed drugs. Because we have previously shown that hyperactivation of the EGFR pathway mimics oncogenic *PIK3CA *mutations [64], our results would suggest that breast cancers with mutant *PIK3CA *and AR expression would have a favorable therapeutic response to AR ligand binding. By contrast, molecular apocrine tumors may benefit from dual MEK and AR inhibition as previously reported [18], and similar results may also apply to HER2-positive/AR-positive breast cancers. Further studies to test these hypotheses may allow for the selection of those patients with breast cancer who will have the highest likelihood of responding to AR-targeted therapies.

## Conclusions

In this study, we constructed new models for AR signaling in human breast epithelial cells. We found that activation of the MAPK pathway by either EGFR or AR signaling leads to cellular proliferation, whereas simultaneous input by both EGFR and AR leads to further increases in MAPK activation and cellular arrest. These findings help elucidate past observations of disparate growth responses to AR ligand binding in various human breast cancer cell lines. Additionally, methods to quantify activation of the MAPK pathway on human tissues may allow for the development of predictive markers of AR signaling that leads to a growth proliferative versus inhibitory phenotype. Moreover, we found that p21 is essential for mediating AR signaling in human breast epithelial cells, regardless of the growth response to AR ligand. Thus, AR expression in conjunction with the presence or absence of p21 may also be useful for predicting sensitivity versus resistance to AR-directed therapies. Significantly, our system provides an ideal model for further study. The use of ARIBE cell lines will help reveal genes and pathways that are crucial for mediating these growth effects, and potentially identify additional predictors of response to AR ligands, thereby accelerating the development of drugs targeting AR for breast cancer therapy.

## Abbreviations

AR: androgen receptor; ARIBE: Androgen Receptor In Breast Epithelium; DAPI: 4'-6-diamidino-2-phenylindole; DMEM: Dulbecco's modified Eagle's medium; DMSO: dimethyl sulfoxide; EGF: epidermal growth factor; EGFR: epidermal growth factor receptor; ERα: estrogen receptor-α; ERK: extracellular signal-regulated kinase; FCS: fetal calf serum; GAPDH: glyceraldehyde 3-phosphate dehydrogenase; MAPK: mitogen-activated protein kinase; PR: progesterone receptor; PVDF: polyvinylidine fluoride; qPCR: quantitative PCR; RNAi: RNA interference; SSC: saline sodium citrate; siRNA: small interfering RNA; TBP: TATA binding protein.

## Competing interests

B.H.P. has received previous research funding from GlaxoSmithKline (GSK) although none of the studies reported here were supported by GSK. B.H.P. is a consultant for GSK and is on the scientific advisory board for Horizon Discovery, LTD and is entitled to payments for these services. These arrangements are managed according to the Johns Hopkins University conflict of interest policy. All of the other authors declare that they have no competing interests.

## Authors' contributions

JP Garay, BK, AMA, MJH, DPC, DJ, AW, PJM, AM, AMD, PA, and BHP designed the experiments; JP Garay, JP Gustin, HK, YK, MM, GMW, CASB, AT, AM, GL and AS performed the experiments; JP Garay, BK, AMA, DPC, MJH, DJ, CASB, PA, AMA and BHP analyzed the data; and JP Garay and BHP wrote the paper. All authors read and approved the final manuscript.

## Supplementary Material

Additional file 1 Supplementary Figures 1 to 8, Tables 1 and 2. Supplementary figures (eight) and tables (two). Click here for file
