# Supplementary material for: The growth response to androgen receptor signaling in ERα-negative human breast cells is dependent on p21 and mediated by MAPK activation
Source: Breast Cancer Res. 2012 Feb 9;14(1):R27. doi: 10.1186/bcr3112 (PMC3496145; doi:10.1186/bcr3112)
Supplement: Additional file 1 — Supplementary Figures 1 to 8, Tables 1 and 2. Supplementary figures (eight) and tables (two). [file bcr3112-S1.PDF]

Supplementary Figure 1.

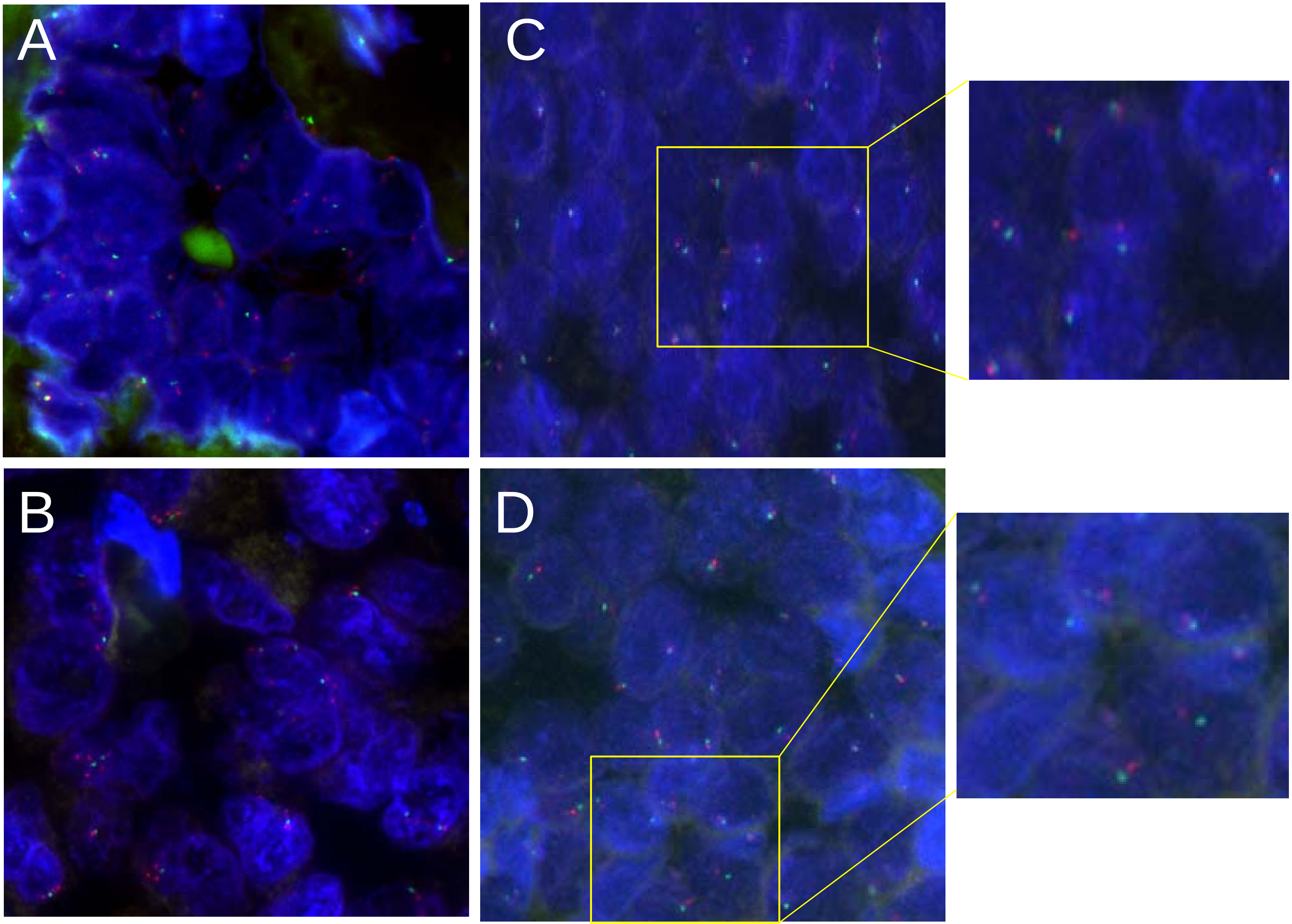

Supplementary Figure 2.

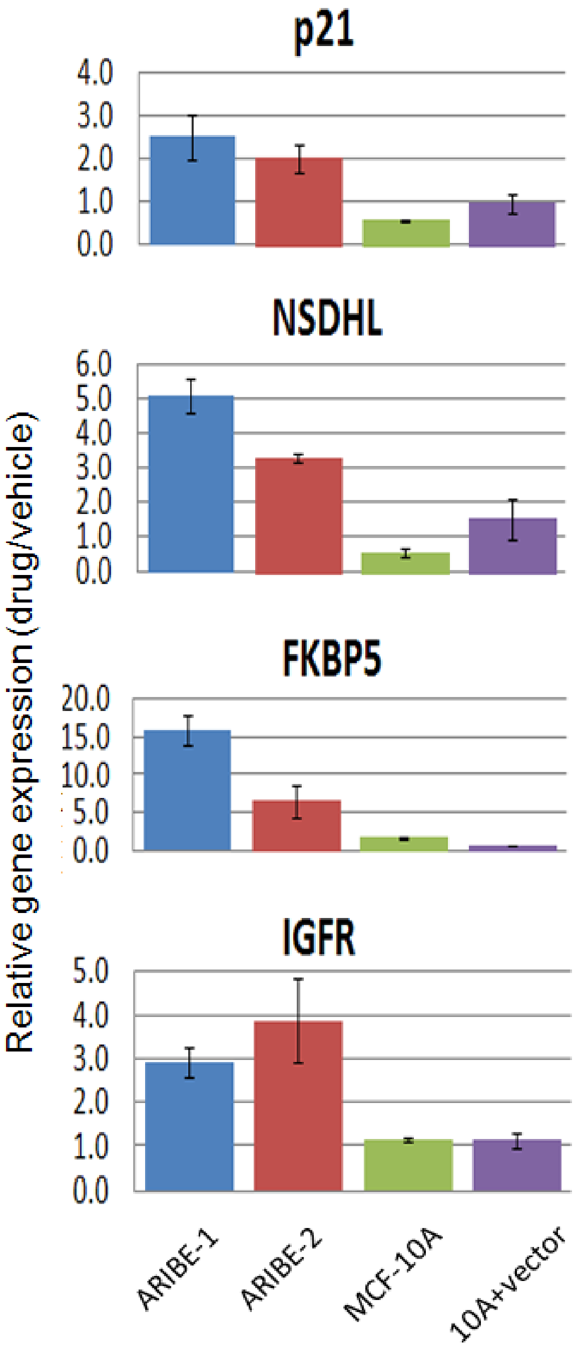

Supplementary Figure 3.

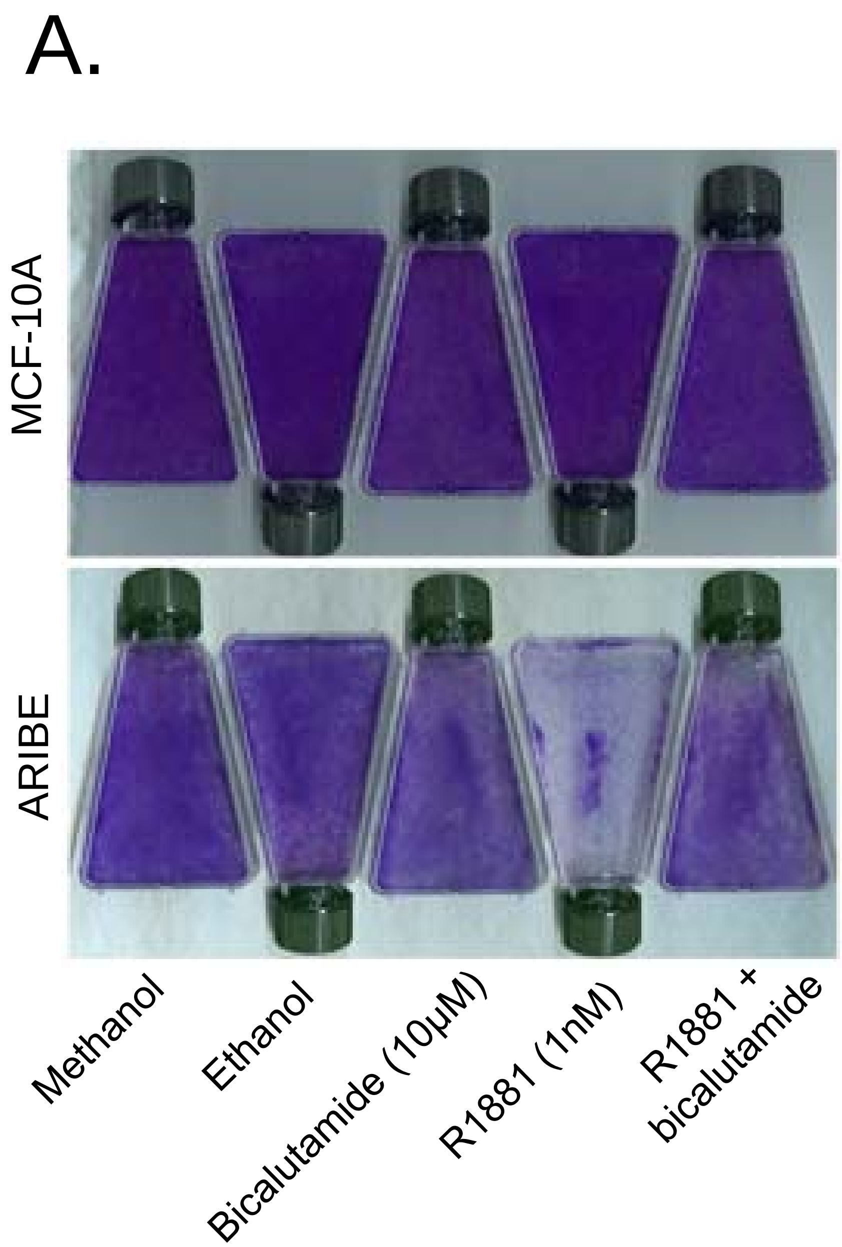

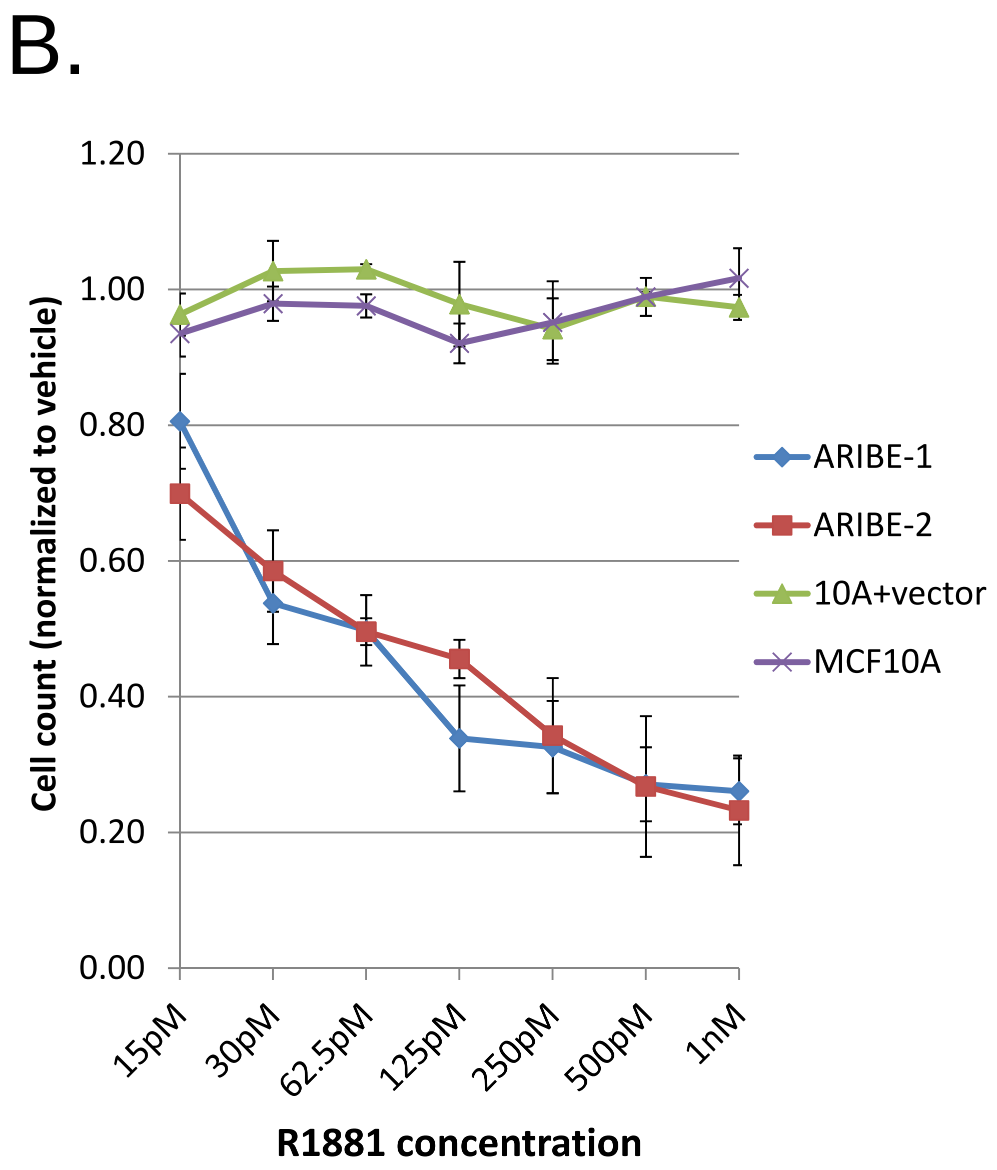

Supplementary Figure 4.

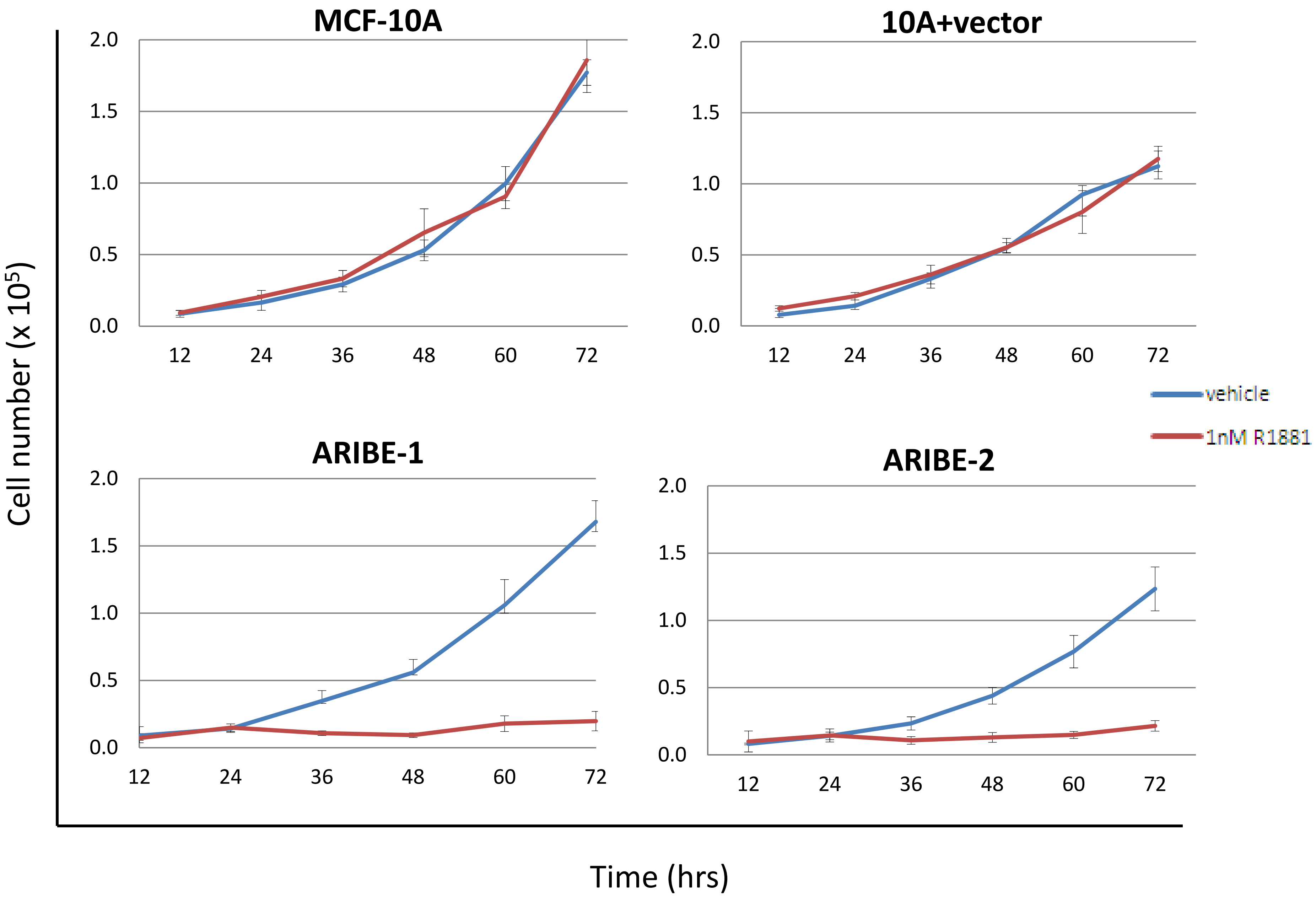

Supplementary Figure 5.

A.

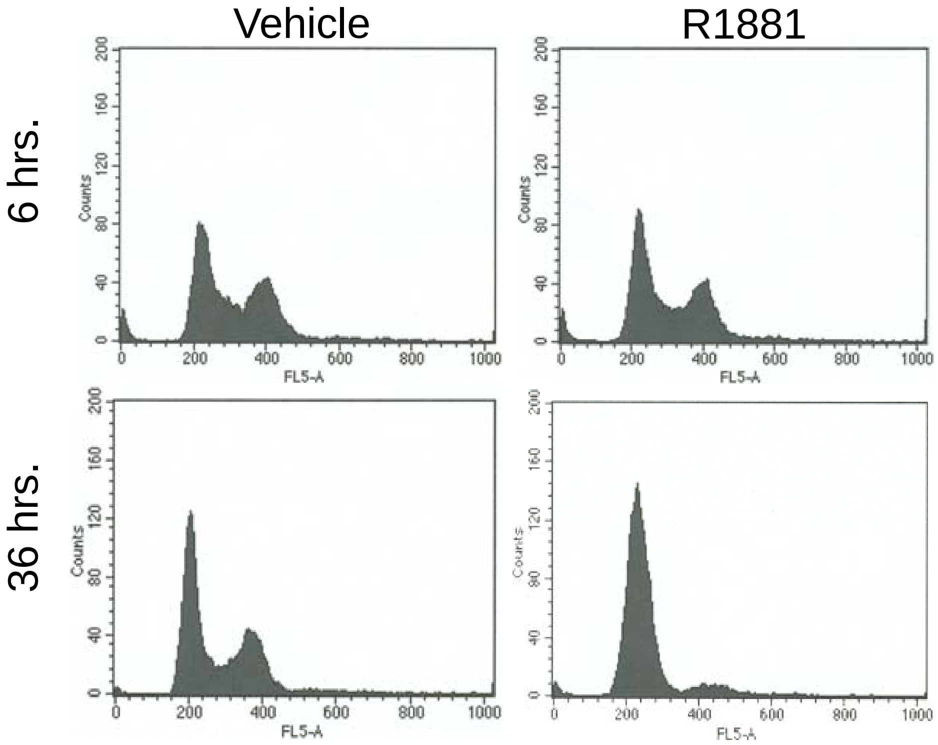

B.

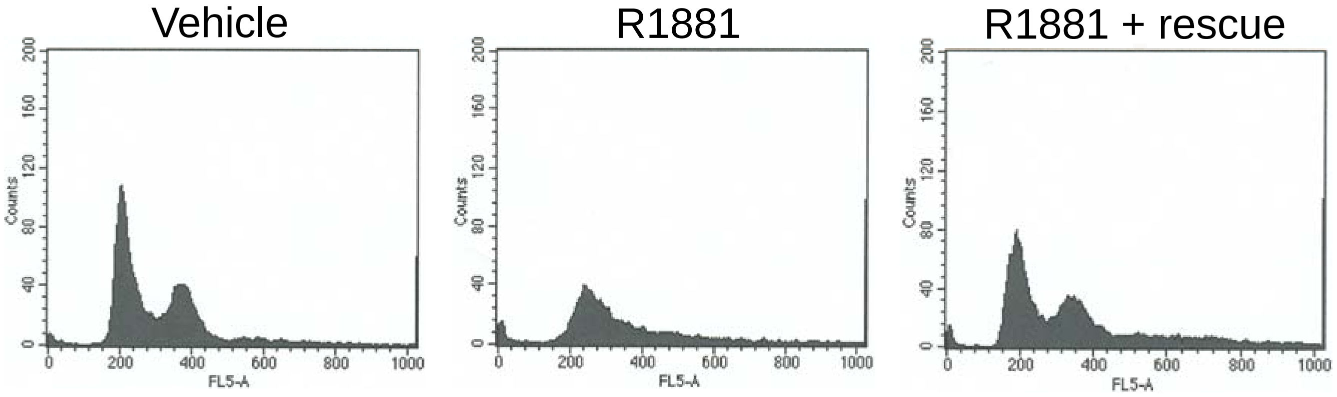

Supplementary Figure 6.

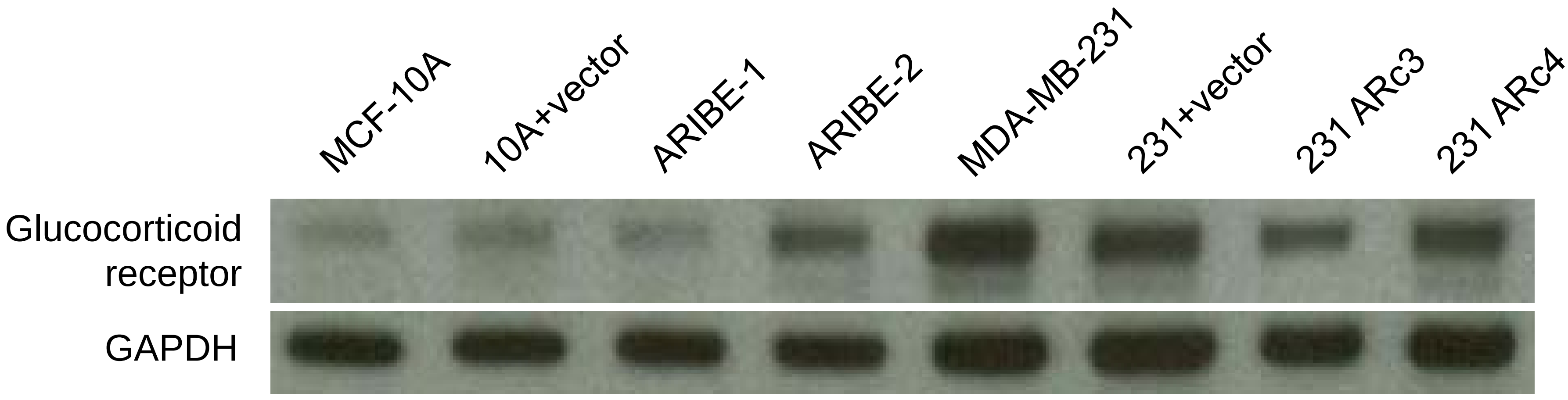

Supplementary Figure 7.

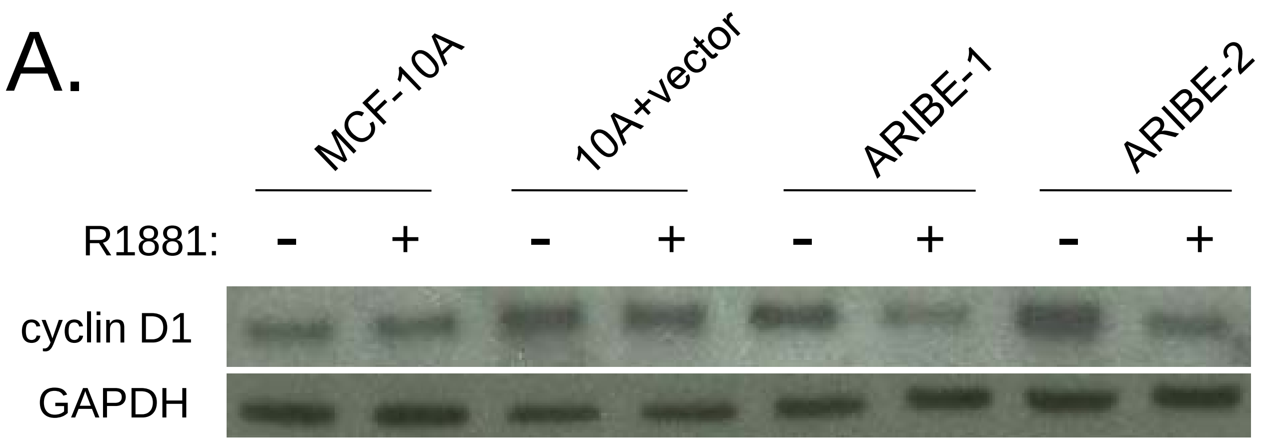

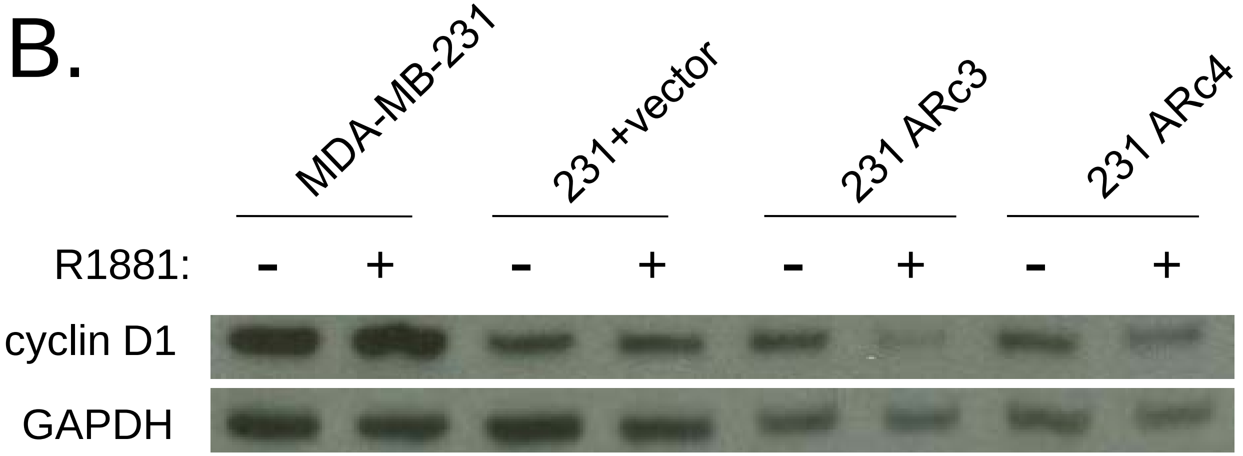

Supplementary Figure 8.

A.

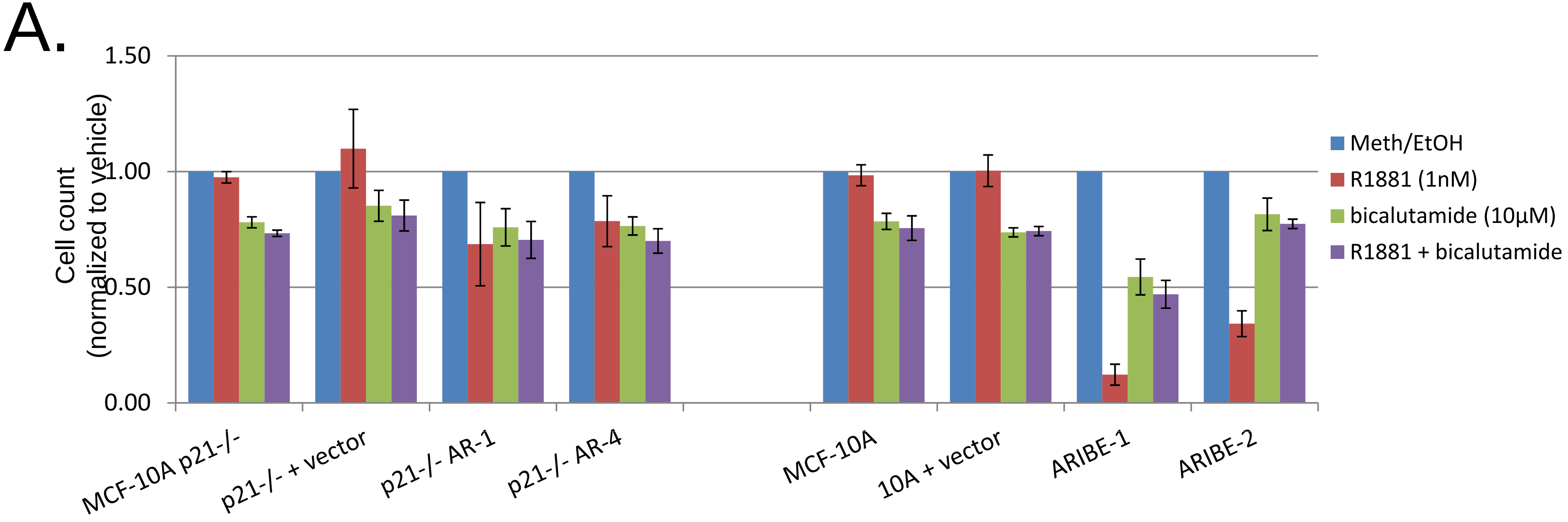

B.

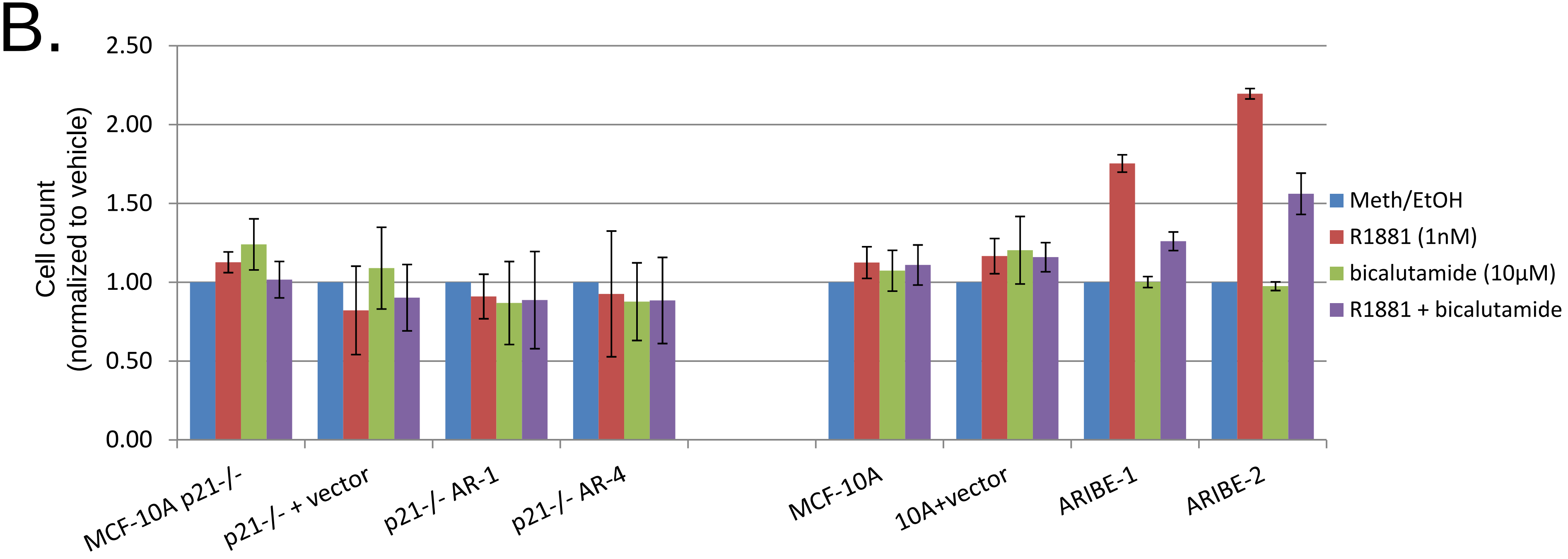

Supplementary Figure Legends

Supplementary Figure 1. Fluorescent *In Situ* Hybridization to examine AR amplification in primary breast cancer biopsies.

A tissue microarray consisting of 99 cores of primary breast cancer tissue and normal tissue was stained for AR (red), X centromere (green), and cell nuclei using DAPI (blue). Fluorescent spots were counted by a pathologist and ratio of AR counts to X centromere counts was calculated.

(A) Sample of normal tissue serving as negative control for AR amplification.

(B) Sample of cell line E006AA, which has a reported AR amplification, serving as positive control for AR amplification.

(C, D) Samples of ductal carcinoma in situ and invasive ductal carcinoma showing no amplification of AR.

Supplementary Figure 2. Expression of AR induced genes at 24 hours. cDNA was made from RNA of cells treated with 1nM R1881 or vehicle for 24 hours. Quantitative real time PCR using SYBR green was performed on triplicate samples of each cell line using intron-spanning primers for each of four androgen response genes. All cycle threshold numbers were normalized to a control gene, TATA binding protein (TBP). Ratio is expression in cells treated with drug versus vehicle. Error bars represent the standard deviation of four independent experiments. In both ARIBE lines, induction of all genes after drug treatment was significant compared to control cell lines by one-way ANOVA ($p<0.05$)

Supplementary Figure 3. Response of ARIBE cells to androgen.

(A) Cell proliferation of MCF-10A parental cells and one representative ARIBE clone. Cells were seeded in flasks at a density of 10^4 cells and treated with vehicle (methanol or ethanol for bicalutamide or R1881, respectively), 10 µM of the AR antagonist bicalutamide, 1 nM of the AR agonist R1881, or the combination of R1881 and bicalutamide. After 3 days flasks were stained with crystal violet.

(B) Two control cells not expressing AR (MCF-10A and 10A+vector) and two ARIBE clones were treated with serial dilutions of R1881. Cells were counted after 3 days of treatment and normalized to count values of cells treated with vehicle. Error bars represent the standard deviation of 3 independent cell counts.

Supplementary Figure 4. Time course of ARIBE response to AR ligand.

Control cells (MCF-10A and 10A+vector) and two ARIBE clones were grown in the presence of EGF and 1 nM R1881 or vehicle. Cells were counted every 12 hours for a period of 72 hours. Error bars represent the standard deviation of 3 independent cell counts.

Supplementary Figure 5. Cell cycle analysis of ARIBE cells treated with R1881.

(A) ARIBE cells were grown in the presence of 1 nM R1881 or vehicle for 6 hours or 36 hours. Cells were harvested and analyzed for cell proliferation using Hoescht stain and FACS. Percent of cells in each phase of the cell cycle (G1/G0, S, and G2/M) was determined using Modfit LT software.

(B) ARIBE cells were grown in the presence of 1 nM R1881 for 48 hours (middle panel) and show arrest in G1/G0 compared to cells treated with vehicle for 48 hours (left panel). Cells arrested in G1/G0 were then washed and replenished with fresh medium not containing R1881 for 48 hours. These cells re-entered the cell cycle and a profile similar to control was restored (right panel).

Supplementary Figure 6. Glucocorticoid receptor expression in AR transgenic cell lines and controls.

Cell lines stably expressing the AR transgene as well as control cell lines were probed for glucocorticoid receptor expression by western blotting. Parental MDA-MB-231 cells served as an internal positive control for antibody binding as recommended by the manufacturer. An antibody for GAPDH was used as a loading control.

Supplementary Figure 7. Downregulation of cyclin D1 expression upon AR ligand binding in the presence of MAP kinase signaling.

(A) Control cells (MCF-10A and 10A+vector) and ARIBE cells were cultured under normal propagation conditions and treated with either vehicle or 1 nM R1881 for 48 hours. Whole cell lysates were probed for expression of cyclin D1. An antibody for GAPDH was used as a loading control.

(B) Control cells (MDA-MB-231 and 231+vector) and MDA-MB-231 cells expressing AR (ARc3 and ARc4) were treated with either vehicle or 1 nM R1881 for 48 hours. Whole cell lysates were probed for expression of cyclin D1. An antibody for GAPDH was used as a loading control.

Supplementary Figure 8. p21 mediates growth effects of AR ligand in MCF-10A cells.

(A) p21-/- (left half) and p21-wildtype (right half) cells were cultured in conditions of 20 ng/mL of EGF and treated with vehicle, 10 μM bicalutamide, 1 nM R1881, or the combination of R1881 and bicalutamide. Cells were counted after 4 days of treatment and normalized to values of cells counted on the day of drug addition (day 0). Error bars represent the standard deviation of the mean from three independent cell counts. The growth difference between p21-wildtype AR-expressing cells (ARIBE-1 and ARIBE-2) treated with R1881 and R1881+bicalutamide is statistically significant by Student’s two-tailed t test ($p<0.001$). No significant difference was found in growth of p21-/- AR-expressing cells treated with R1881.

(B) Cells were cultured in the absence of EGF and treated with drugs as above. Cells were counted after 8 days of treatment and normalized to values of cells counted on the day of drug addition (day 0). Error bars represent the standard deviation of the mean from three independent cell counts. The growth difference between p21-wildtype AR-expressing cells (ARIBE-1 and ARIBE-2) treated with R1881 and R1881+bicalutamide is statistically significant by Student’s two-tailed t test ($p<0.01$). No significant difference was found in growth of p21-/- AR-expressing cells treated with R1881.

Supplementary Table 1- Primers used for this study

**primer**	**sequence (5'->3')**	**use**
AR-F	ATGGAAGTGCAGTTAGGGCTG	clone cDNA of AR
AR-R	TCACTGGGTGTGGAAATAGATGGGC	(BamHI site was added to primers, not shown in this sequence)
IGFR-F	TGCTGACCTCTGTTACCTCTCC	quantitative real time PCR
IGFR-R	CCGGCATAGTAGTAGTGGCG	
FKBP5-F	GAATGGTGAGGAAACGCCG	quantitative real time PCR
FKBP5-R	AACATCCTTCCACCACAGCG	
NSDHL-F	CTATCCCTGCTGGTGATGGT	quantitative real time PCR
NSDHL-R	GGCTCTCTCGCAGCTGTAGT	
p21-F	CGGATTCGCCGAGGCACCG	quantitative real time PCR
p21-R	CCATTAGCGCATCACAGTCG	

Supplementary Table 2. Protein expression values of quantified western blot samples

		Sample	Protein Expression Value	Ratio
**Fig. 1A**		ARIBE-1	1.11	
		ARIBE-2	1.18	
		MCF-10A	0.05	
		10A+vector	0.03	
		MDA-MB-453	0.42	
		LNCaP	0.82	
**Fig. 3**	EGF	MCF-10A	0.96	(phosphorylated Erk,R1881/total Erk,R1881) / (phosphorylated Erk,EtOH/total Erk,EtOH)
	EGF	10A+vector	1.05	(phosphorylated Erk,R1881/total Erk,R1881) / (phosphorylated Erk,EtOH/total Erk,EtOH)
	EGF	ARIBE-1	3.21	(phosphorylated Erk,R1881/total Erk,R1881) / (phosphorylated Erk,EtOH/total Erk,EtOH)
	EGF	ARIBE-2	3.96	(phosphorylated Erk,R1881/total Erk,R1881) / (phosphorylated Erk,EtOH/total Erk,EtOH)
	no EGF	MCF-10A	0.05	(phosphorylated Erk,R1881/total Erk,R1881) / (phosphorylated Erk,EtOH/total Erk,EtOH)
	no EGF	10A+vector	0.06	(phosphorylated Erk,R1881/total Erk,R1881) / (phosphorylated Erk,EtOH/total Erk,EtOH)
	no EGF	ARIBE-1	16.00	(phosphorylated Erk,R1881/total Erk,R1881) / (phosphorylated Erk,EtOH/total Erk,EtOH)
	no EGF	ARIBE-2	18.00	(phosphorylated Erk,R1881/total Erk,R1881) / (phosphorylated Erk,EtOH/total Erk,EtOH)
**Fig. 4A**		231 ARc3	0.90	
		231 ARc4	0.94	
		231+vector	0.00	
		MDA-MB-453	0.56	
		LNCaP	1.04	
**Fig. 5A**		MCF-10A	1.12	p21,R1881 / p21,EtOH
		10A+vector	1.09	p21,R1881 / p21,EtOH
		ARIBE-1	2.83	p21,R1881 / p21,EtOH
		ARIBE-2	3.69	p21,R1881 / p21,EtOH
**Fig. 5B**		MDA-MB-231	1.09	p21,R1881 / p21,EtOH
		231+vector	0.98	p21,R1881 / p21,EtOH
		231 ARc3	4.82	p21,R1881 / p21,EtOH
		231 ARc4	16.30	p21,R1881 / p21,EtOH
**Fig. 6A**		ARIBE-1 siRNA #1	0.02	p21,sample / p21,ARIBE-1 no siRNA
		ARIBE-1 siRNA #3	0.03	p21,sample / p21,ARIBE-1 no siRNA
		ARIBE-1 control siRNA	1.40	p21,sample / p21,ARIBE-1 no siRNA

**Fig. 6B**		ARIBE-2 siRNA #1	0.03	p21,sample / p21,ARIBE-2 no siRNA
		ARIBE-2 siRNA #2	0.03	p21,sample / p21,ARIBE-2 no siRNA
		ARIBE-2 control siRNA	1.36	p21,sample / p21,ARIBE-2 no siRNA
**Fig. 7A**		p21-/- AR-1	1.11	
		p21-/- AR-4	1.37	
		MCF10A p21-/-	0.09	
		p21-/- + vector	0.02	
		ARIBE-1	0.99	
		ARIBE-2	0.90	
**Fig. 8**	EGF	MCF10A p21-/-	1.11	(phosphorylated Erk,R1881/total Erk,R1881) / (phosphorylated Erk,EtOH/total Erk,EtOH)
		p21-/- + vector	0.93	(phosphorylated Erk,R1881/total Erk,R1881) / (phosphorylated Erk,EtOH/total Erk,EtOH)
		p21-/- AR-1	0.97	(phosphorylated Erk,R1881/total Erk,R1881) / (phosphorylated Erk,EtOH/total Erk,EtOH)
		p21-/- AR-4	1.19	(phosphorylated Erk,R1881/total Erk,R1881) / (phosphorylated Erk,EtOH/total Erk,EtOH)
	no EGF	MCF10A p21-/-	0.0	(phosphorylated Erk,R1881/total Erk,R1881) / (phosphorylated Erk,EtOH/total Erk,EtOH)
		p21-/- + vector	0.0	(phosphorylated Erk,R1881/total Erk,R1881) / (phosphorylated Erk,EtOH/total Erk,EtOH)
		p21-/- AR-1	0.0	(phosphorylated Erk,R1881/total Erk,R1881) / (phosphorylated Erk,EtOH/total Erk,EtOH)
		p21-/- AR-4	0.0	(phosphorylated Erk,R1881/total Erk,R1881) / (phosphorylated Erk,EtOH/total Erk,EtOH)
**Fig. S6**		MCF-10A	0.19	
		10A+vector	0.29	
		ARIBE-1	0.23	
		ARIBE-2	0.38	
		MDA-MB-231	0.89	
		231+vector	0.82	
		231 ARc3	0.75	
		231 ARc4	0.78	
**Fig. S7A**		MCF10A	1.11	cyclin D1,R1881 / cyclin D1,EtOH
		10A+vector	1.05	cyclin D1,R1881 / cyclin D1,EtOH
		ARIBE-1	0.63	cyclin D1,R1881 / cyclin D1,EtOH
		ARIBE-2	0.58	cyclin D1,R1881 / cyclin D1:EtOH
**Fig. S7B**		MDA-MB-231	1.23	cyclin D1,R1881 / cyclin D1,EtOH
		231+vector	1.19	cyclin D1,R1881 / cyclin D1,EtOH
		231 ARc3	0.37	cyclin D1,R1881 / cyclin D1,EtOH
		231 ARc4	0.68	cyclin D1,R1881 / cyclin D1,EtOH

All samples were normalized to a corresponding GAPDH loading control. For experiments comparing various samples, the ratio used to calculate the given value after normalization to GAPDH is included in the table. For Fig. 6, results were normalized to no siRNA controls. Results are representative of multiple independent blots.
